# MicroRNAs as Biomarkers for Coronary Artery Disease Related to Type 2 Diabetes Mellitus—From Pathogenesis to Potential Clinical Application

**DOI:** 10.3390/ijms24010616

**Published:** 2022-12-29

**Authors:** Joanna Szydełko, Beata Matyjaszek-Matuszek

**Affiliations:** Department of Endocrinology, Diabetology and Metabolic Diseases, Medical University of Lublin, Jaczewskiego 8, 20-090 Lublin, Poland

**Keywords:** microRNA, type 2 diabetes mellitus, coronary artery disease, atherosclerosis, biomarker, chronic inflammation

## Abstract

Type 2 diabetes mellitus (T2DM) is a chronic metabolic disease with still growing incidence among adults and young people worldwide. Patients with T2DM are more susceptible to developing coronary artery disease (CAD) than non-diabetic individuals. The currently used diagnostic methods do not ensure the detection of CAD at an early stage. Thus, extensive research on non-invasive, blood-based biomarkers is necessary to avoid life-threatening events. MicroRNAs (miRNAs) are small, endogenous, non-coding RNAs that are stable in human body fluids and easily detectable. A number of reports have highlighted that the aberrant expression of miRNAs may impair the diversity of signaling pathways underlying the pathophysiology of atherosclerosis, which is a key player linking T2DM with CAD. The preclinical evidence suggests the atheroprotective and atherogenic influence of miRNAs on every step of T2DM-induced atherogenesis, including endothelial dysfunction, endothelial to mesenchymal transition, macrophage activation, vascular smooth muscle cells proliferation/migration, platelet hyperactivity, and calcification. Among the 122 analyzed miRNAs, 14 top miRNAs appear to be the most consistently dysregulated in T2DM and CAD, whereas 10 miRNAs are altered in T2DM, CAD, and T2DM-CAD patients. This up-to-date overview aims to discuss the role of miRNAs in the development of diabetic CAD, emphasizing their potential clinical usefulness as novel, non-invasive biomarkers and therapeutic targets for T2DM individuals with a predisposition to undergo CAD.

## 1. Introduction

Type 2 diabetes mellitus (T2DM) is one of the most common metabolic disorders worldwide, and its prevalence has been growing among adolescents and young adults at an alarming rate over the past three to four decades [1,2,3]. According to the International Diabetes Federation, 537 million people suffered from diabetes mellitus in 2021, and this number is expected to rise up to 783 million by 2045 [4]. It is estimated that T2DM accounts for approximately 90–95% of all cases of diabetes mellitus, depending on different ethnicity [3,5]. However, it is worth underlining that at least one-third to one-half of people living with T2DM are undiagnosed and untreated [6,7].

T2DM is a chronic, low-grade inflammatory disease characterized by hyperglycemia that results from a progressive beta-cell impairment, insulin secretion deficiency, and concomitant insulin resistance [8]. Long-term exposure to uncontrolled hyperglycemia leads to the development of various micro- and macrovascular complications, including diabetic retinopathy, nephropathy, peripheral neuropathy, and diabetic foot syndrome, as well as cardiovascular and cerebrovascular diseases [9,10]. Initially, the clinical symptoms of the disease may be very subtle, which most likely results in a prolonged diagnostic delay of an average of 5–6 years after the onset of the first signs, therefore the presence of at least one vascular complication can be observed even in newly diagnosed T2DM cases [7,10]. A large cross-sectional study in Denmark revealed that 35% of previously undiagnosed T2DM individuals had complications at the time of diagnosis, of whom 17% had macrovascular ones, with the greater risk of ischemic heart disease occurring in 15% of patients, 12% had microvascular, whereas 6% of participants presented both micro- and macrovascular complications [10].

It should be emphasized that patients with T2DM are two to four times more susceptible to developing coronary artery disease (CAD) than non-diabetic individuals, but on the other hand, the prevalence of diabetes mellitus in CAD patients is up to 40%, and another 6% of them will develop ‘silent’ diabetes in the next few years [11,12,13,14,15]. Anyway, more than half of T2DM individuals die of cardiovascular causes [11,16,17]. Both T2DM and CAD are complex, multifactorial diseases with genetic, epigenetic, and environmental backgrounds [8,18]. Chronic hyperglycemia and insulin resistance stimulate oxidative stress and inflammation, and thus they initiate endothelial dysfunction and contribute to accelerated atherosclerosis, which is the main driver of CAD development [19]. Moreover, persistently high blood glucose levels lead to abnormal activation of platelet, vascular calcification, and deterioration of atherogenic dyslipidemia, that further promote the atherosclerotic process and increase the risk of thrombus formation [19,20]. Therefore, the identification of patients at an early stage of the disease is critical for prevention and adequate treatment.

In recent years, microRNAs (miRNAs, miRs), belonging to an ever-growing family of highly conserved, naturally occurring non-coding RNA (ncRNA) molecules, have emerged as key players in different physiological and pathological processes, such as cell proliferation, differentiation, and apoptosis [21]. Dysregulation of miRNA expression signatures is closely associated with numerous diseases, including diabetes mellitus and cardiovascular disorders. Thus they are considered novel promising biomarkers [22,23,24,25].

The present review is aimed to discuss the role of miRNAs in the pathogenesis of diabetic CAD, moving from preclinical to clinical studies, and to highlight their potential usefulness as early, noninvasive biomarkers and therapeutic targets for CAD in patients with T2DM.

## 2. Biology of MicroRNAs

**Figure 1:**
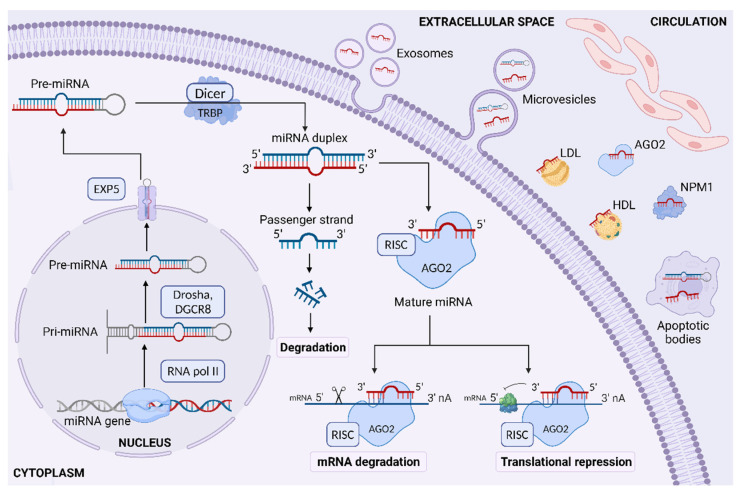
Canonical pathway of miRNA biogenesis in humans. miRNA genes are transcribed by RNA pol II into pri-miRNA transcripts in the nucleus. Pri-miRNA is processed to pre-miRNA with the Drosha enzyme, and then it is transported to the cytoplasm by EXP5. Dicer endonuclease cleaves it to a short miRNA duplex. Mature miRNA, as a part of the RISC complex, binds with the 3′-untranslated region of target mRNA on different complementarity leading to mRNA degradation or translational repression. Both pre-miRNA and mature miRNA can be released passively or actively into the extracellular space in apoptotic bodies, exosomes, microvesicles, or in the complexes with AGO2, NPM1, HDL, and LDL. miRNA—microRNA; RNA pol II—RNA polymerase II; Pri-miRNA—primary miRNA; Pre-miRNA—precursor miRNA; DGCR8—DiGeorge syndrome Critical Region 8; EXP5—exportin 5; TRBP—trans-activation response RNA binding protein; AGO2—Argonaute 2; RISC—RNA-induced silencing complex; mRNA—messenger RNA; NPM1—nucleophosmin 1; HDL—high-density lipoprotein; LDL—low-density lipoprotein. Created with BioRender.com.

Regulatory ncRNAs could be classified into several types based on their average size involving small ncRNAs (sncRNAs) with less than 200 nucleotides (miRNA, small interfering RNA; siRNA, PIWI-interacting RNA; piRNA), long ncRNAs (lncRNAs) with more than 200 nucleotides, and circular RNAs (circRNAs) with a special closed-loop structure [26]. miRNAs are a dominating class of small endogenous, single-stranded ncRNAs, ranging from 18 to 25 nucleotides in length, that posttranscriptionally regulate gene expression through different pathways [27,28]. The biogenesis of miRNA involves multi-step processes occurring both in the nucleus and in the cytoplasm [28,29]. In the first step of this molecular pathway, miRNA-encoding sequences located in intergenic (40%) or intragenic (60%) regions of the genome are transcribed mostly by RNA polymerase II, which leads to the synthesis of primary miRNA (pri-miRNA) transcript with a 5′-cap and a 3′ poly-A tail in the nucleus [28,29,30]. Pri-miRNAs are long, double-stranded products consisting of approximately 1000 nucleotides having a hairpin-like stem-loop-like structure flanked by single-stranded RNA ends [29]. The nuclear microprocessor protein complex comprised of Drosha, an RNase type III enzyme, and its co-factor known as the double-stranded RNA-binding protein DiGeorge syndrome Critical Region 8 (DGCR8), cleaves pri-miRNA into 70–90 nucleotide precursor miRNA (pre-miRNA), which is then transported through the nuclear membrane to the cytoplasm by the Exportin 5 (EXP5, encoded by *XPO5*)—RanGTP system [28,29]. In the cytoplasm, pre-miRNAs are further processed by RNase III enzyme Dicer/trans-activation response RNA binding protein (TRBP, encoded by *TARBP2*) complex to generate shorter, about 22-nucleotide base pair miRNA duplex (miRNA-3p/miRNA-5p) [28,29]. In the last step of this process, miRNA duplex is loaded into the pre-RNA-induced silencing complex (pre-RISC) containing Argonaute proteins (AGO1-4, in humans), of which only AGO2 has the target cleavage activity [28,29,31]. Subsequently, one strand of miRNA duplex, called guide strand or miRNA, remains bound to AGO2 as mature miRNA, whereas another one, passenger strand or miRNA*, is degraded, and thus mature RISC is generated [28,29]. It is worth underlining that the formation of miRNA/pre-RISC complex is an active process that requires an ATP-dependent mechanism involving the heat shock cognate 70 (HSC70)—heat shock protein 90 (HSP90) chaperone complex to mediate conformational changes of AGO proteins and allow them to bind stiff double-stranded miRNA, while the release of the passenger strand is ATP-independent [28,29]. Mature miRNA targets specific messenger RNA (mRNA) by base pairing in the 3′ untranslated regions (3′ UTR) of these transcripts, and in this way, it induces posttranscriptional gene silencing through translational repression and/or mRNA degradation [28,32]. In humans, inhibition of protein synthesis is mediated through perfect or imperfect complementarity in the ‘seed’ region of miRNA composed of 2–8 nucleotides and target mRNA that results in mRNA cleavage or translational repression, respectively [28,32]. Additionally, the degradation of miRNA targets can be accelerated by mRNA deadenylation and decapping via glycine-tryptophan protein of 182 kDa (GW182) interaction with AGO2 [28,33]. Particular steps involved in the biogenesis and functions of miRNAs in humans are shown in Figure 1.

Apart from the above-mentioned canonical pathway of miRNA synthesis, there are some alternative ways of its maturation, including inter alia Drosha/DGCR8-independent and Dicer-independent pathways, which generate about 1% of conserved miRNAs [28,29]. The well-recognized ‘mirtron’ route is responsible for transforming an unconventional class of intragenic miRNAs, so-called mirtrons, escaping from Drosha/DGCR8-mediated process into pre-miRNAs by splicing enzymes (spliceosome) [28,29,34]. In the next step, mirtron-derived pre-miRNAs are exported to the cytoplasm by EXP5 and cleaved by Dicer, similar to the canonical pathway [28,29,34]. In turn, Dicer-mediated processing is only bypassed in the case of pre-miR-451 hairpins, resulting from Drosha cleavage, that are too short (30 nucleotides in length) to be processed by Dicer, and their maturation relies on the catalytic activity of AGO2 [28,29,34,35]. These short substrates are then sliced by AGO2 in the middle of their 3p strand and follow 3′-5′ trimming of the 5p strand to complete their maturation [28,29,34,35].

Although the majority of synthesized miRNAs are found intracellularly, a significant amount appears in the extracellular space [36]. miRNAs are released into the circulation not only passively from damaged cells due to apoptosis or necrosis, but they can also be actively secreted in several extracellular vesicles (EVs), including exosomes (40–100 nm), microvesicles (100–1000 nm) and apoptotic bodies (50–5000 nm) [36,37]. In addition, about 90% of circulating miRNAs form complexes with RNA-binding proteins, including AGO2, nucleophosmin 1 (NPM1) proteins, and lipoproteins, which protect them from RNase-dependent degradation [36].

The first miRNA, lineage-4 (lin-4), was discovered in 1993 by Victor Ambros and colleagues, Rosalind Lee and Rhonda Feinbaum in *Caenorhabditis elegans*, followed by the first human miRNA lethal-7 (let-7), which was identified 7 years later [38,39]. Since then, more than 2600 human miRNAs have been described according to the online database miRBase (www.mirbase.org, version 22), and the list is still expanding [40]. Although miRNAs have been considered unimportant additions to the transcriptome over decades, currently, a growing body of evidence indicates that they are critical modulators of gene expression [41]. What is interesting, one miRNA may silence from 100 to even 200 genes, and at the same time, multiple miRNAs are able to converge on a single protein-coding gene target [42,43]. According to the data collected in the latest version of miRTarBase (http://miRTarBase.cuhk.edu.cn/, accessed on 24 November 2022), approximately 4,475,477 validated human miRNA-target interactions are known [44]. It is worth emphasizing that miRNAs regulate at least one-third of all genes within the human genome, although the biological functions of only 3.6% of them were confirmed in the experiments [45,46]. miRNAs are involved in a wide range of processes and signaling pathways in different cells and tissues, as well as their disturbed expression profile is observed in many diseases [21,45]. Recent studies underscore their role in the pathogenesis of atherosclerosis under a condition of long-term hyperglycemia that, in consequence, leads to the development of CAD in T2DM [19,47].

## 3. Type 2 Diabetes Mellitus (T2DM), Atherosclerosis and Coronary Artery Disease (CAD)—The Vicious Circle Paradigm

Atherosclerosis is a chronic inflammatory disease of the inner wall of large- or medium-sized arteries caused by endothelial injury and subendothelial accumulation of lipid, extracellular matrix proteins, and calcium deposits, forming fibroinflammatory lipid plaques, particularly at sites of disturbed blood flow [19]. The formation of atheroma in one or more coronary arteries can narrow the lumen of the blood vessel, which leads to ischemia and metabolic changes in the alimented tissues [19,48]. Furthermore, the subsequent rupture of the coronary artery atherosclerotic plaque may result in coronary thrombus and its inevitable consequences, including unstable angina and acute myocardial infarction [48].

Clinical and experimental data have strongly indicated that the presence of T2DM is associated with a diffuse, more severe, and rapidly progressive form of atherosclerosis. Thereby atherogenesis is a key player linking T2DM with CAD [49,50]. Chronic hyperglycemia and insulin resistance mediate endothelial injury via several pathological pathways involved in the regulation of metabolism-immune system interplay [19,50]. Oxidative stress and chronic low-grade inflammation have been shown to be the main triggers of endothelial dysfunction in prolonged hyperglycemic conditions [47]. Diabetic atherogenesis is a complex, multi-step process characterized by the infiltration of inflammatory cells, monocyte/macrophage activation, vascular smooth muscle cell (VSMC) differentiation, and platelet hyperactivity, along with dysfunctional endothelial cells (ECs) and endothelial-to-mesenchymal transition (EndMT) [8,19,50,51]. Additionally, these events are enhanced by atherogenic dyslipidemia, which commonly accompanies T2DM [19].

The close relationship between T2DM and atherosclerosis is well established, although the epigenetic mechanisms underlying the development of diabetic atherosclerosis are still not completely understood. In the last years, numerous studies have revealed that specific miRNAs are able to modulate almost every step of the atherogenic process from its initiation through progression and, ultimately, thrombotic complications [47,50].

### 3.1. The Role of MicroRNAs in the Initiation of T2DM-Associated Atherosclerosis

#### 3.1.1. MicroRNAs in Chronic Hyperglycemia-Induced Endothelial Dysfunction

Endothelial dysfunction is considered a major contributor to the initiation of diabetic atherosclerosis, and it requires the orchestration of a series of events, including oxidative stress and inflammatory processes [19]. The enhanced levels of reactive oxygen species (ROS) under hyperglycemic conditions are attributable to both overproduction of oxygen-derived radicals and impaired antioxidant defense resulting from a decline in mitochondrial function, increased expression of nicotinamide adenine dinucleotide phosphate (NADPH) oxidase, cyclooxygenase (COX), myeloperoxidase (MPO) as well as decreased expression of superoxide dismutase which then lead to a generation of superoxide anion (O_2_^-^), a precursor of the majority of other ROS [52]. In addition, persistent high glucose levels induce the low-grade inflammation reflected by elevated levels of proinflammatory cytokines and chemokines, including interleukin 1β (IL-1β), interleukin 6 (IL-6), interleukin 18 (IL-18), and tumor necrosis factor α (TNF-α) [19,53]. Simultaneously, increased oxidative stress and inflammation in the hyperglycemic milieu accelerate the nonenzymatic glycoxidation of proteins and lipids that results in advanced glycation end-products (AGEs) formation [19,20]. What is more, a predominant precursor of AGEs is also methylglyoxal (MGO), a side-product of glycolysis which accumulates in the cells under diabetic conditions and contributes to insulin resistance [53,54,55]. Upon AGE binding with its surface receptor (RAGE), multiple intracellular signal transduction pathways are activated, while soluble RAGE (sRAGE), a secretory isoform lacking the transmembrane domain, contributes to the neutralization or removal of circulating ligands by competing with membranous RAGE [20,53,56]. In addition to AGEs, RAGE is able to bind several other proinflammatory particles, including high mobility group box-1 (HMGB1), S100 calcium-binding proteins, and amyloid-β-protein, which provokes EC activation and increased expression of surface adhesion molecules, such as vascular cell adhesion molecule-1 (VCAM-1), intercellular adhesion molecule-1 (ICAM-1), and E-selectin via targeting nuclear factor-kappa B (NF-κB) pathway [19,53,57]. Hence, these actions promote the adhesion and entrance of monocytes/macrophages into the subendothelial space, exaggerate inflammatory response and initiate atherosclerotic plaque formation [19]. Long-lasting AGE/RAGE axis activation inhibits biosynthesis of nitric oxide (NO), a major vasodilator, by endothelial NO synthase (eNOS) downregulation and synchronously enhances the production of endothelium-derived COX-dependent contracting factors (EDCFs) and endothelin-1 (ET-1) [58]. The imbalance between vasodilators and vasoconstrictors in favor of the latter leads to disturbances in vascular tone and endothelial dysfunction [58]. Furthermore, AGE-RAGE interaction increases oxidative stress, endoplasmic reticulum stress, and inflammation in ECs [47,56]. It is worth noting that heightened glucose uptake by ECs intensifies the synthesis of diacylglycerol, which along with increased AGEs, ROS, and inflammatory factors, activate protein kinase C (PKC) signaling. This, in consequence, initiates the c-Jun N-terminal kinase (JNK)/extracellular signal-regulated kinases 1/2 (ERK 1/2)/p38 mitogen-activated protein kinase (MAPK) pathway and NADPH oxidase, whereas it inhibits downstream expression of phosphatidylinositol 3-kinase (PI3K)/Akt [19,47,56]. In this way, PKC is able to modulate the migration and proliferation of ECs, increase oxidative stress and inflammation, as well as inhibit angiogenesis and NO production [19,47].

Numerous miRNAs have been implicated in the regulation of endothelial homeostasis, vascular repair, and angiogenesis. Among them, miR-126 was first identified as an endothelial-specific miRNA that is critical for preserving EC integrity, and its level has been found to be decreased in the diabetic microenvironment [59,60,61,62]. The restored expression of miR-126 may suppress inflammation and ROS production by diminishing the expression of downstream components of HMGB1, including TNF-α, NADPH oxidase activity, and triggering AKT-eNOS pathway in ECs treated by high glucose [59]. In addition, miR-126 overexpression in endothelial progenitor cells (EPCs), which are released from the bone marrow in response to endothelium damage or tissue ischemia, may protect against EPC dysfunction induced by hyperglycemia-associated AGEs and promote their proliferation, migration, and inhibit apoptosis that enables repairing the injured endothelium, and reendothelialization [60,61,62]. It was observed that miR-126 is also able to decrease proinflammatory and oxidative stress markers via its target sprouty-related, EVH1 domain-containing protein 1 (Spred-1) as well as through inducing Ras/ERK/vascular endothelial growth factor (VEGF) and PI3K/Akt/eNOS signaling pathways [60,61,62]. Moreover, the recovering of miR-130a level has been found to exert a protective effect on EPCs by targeting Runx3 and JNK, ERK/VEGF, PI3K/Akt pathways, thus increasing proliferation, migration, and differentiation of EPCs, and reducing their apoptosis [63,64,65].

Another miRNA that has been widely studied in the context of high glucose-induced endothelial dysfunction is miR-21 [66,67,68]. Its expression is markedly enhanced in circulating ECs from diabetic patients and in high glucose-treated ECs [66,67,68]. One of the research has suggested an atheroprotective role of miR-21 on ECs exposed to high glucose conditions, probably by inhibiting the expression of death-domain associated protein (DAXX), a factor related to oxidative stress-induced cell death, although miR-21 by itself did not affect high glucose-induced ROS production in ECs [66]. By contrast, other authors have shown the adverse effects of miR-21 on the endothelium [67,68]. The elevated expression of miR-21 in diabetic conditions leads to increased oxidative stress-mediated endothelial dysfunction by downregulation of antioxidant response genes, specifically, SOD2, which induces the release of large amounts of O_2_^−^ [67]. Anyway, miR-21-3p has been revealed to aggravate the atherosclerotic lesions through the activation of the AGE/RAGE axis and its downstream signaling pathways, including the PKC, MAPK, and NF-κB, that, in consequence, increase ROS generation and intensify inflammatory state reflected by high levels of IL-1β, IL-6, TNF-α, and monocyte chemoattractant protein-1 (MCP-1), also known as chemokine CC-motif ligand 2 (CCL-2) [68]. Paradoxically, miR-21-3p may also be responsible for the degradation of a disintegrin and metalloproteinase domain-containing protein 10 (ADAM10), an enzyme involved in the ectodomain shedding of RAGE, thus leading to reduced sRAGE levels, while no difference was observed in RAGE expression upon miR-21-3p mimicking or inhibition [68].

Notably, the downregulation of miR-214 and miR-190a exert a significant role in MGO-induced endothelial insulin resistance, at least in part, by increasing their specific targets, PH domain leucine-rich repeat protein phosphatase 2 (PHLPP2) levels, a negative regulator of the insulin signaling and GTPase Kirsten Rat Sarcoma Viral Oncogene Homolog (KRAS), respectively [54,55]. Meanwhile, the overexpression of these miRNAs rescues the insulin effect on Akt or insulin receptor substrate 1 (IRS1)/Akt/eNOS pathway, improves NO release in response to insulin, and prevents the hyperactivity of ERK1/2 in MGO-treated ECs [54,55].

Intriguingly, upregulation of miR-185 and miR-320, miRNA related to high glucose-induced metabolic memory, protects against vascular endothelial dysfunction via decreasing RAGE, PKC, and/or VEGFA protein levels, which is connected with suppressed proliferation and angiogenesis capacity of ECs, whereas surprisingly they may inhibit (miR-185) or promote (miR-320) EC apoptosis [69,70].

So far, only miR-200a and miR-200c, out of the miR-200 family, have been studied within the context of ROS-induced diabetic endothelial dysfunction [71,72,73]. miR-200a, which is downregulated in hyperglycemia, is engaged in endothelial antioxidant and anti-inflammatory defense by reducing ROS, lipid peroxide marker, VCAM-1, and MCP-1 levels and driving endothelial NO production [71]. However, miR-200c impairs endothelial function by increasing COX-2 and vasoconstrictors, such as prostaglandin E2, thus limiting endothelium-dependent relaxation, and it may also decrease EC proliferation under hyperglycemia-induced oxidative stress [72,73]. Likewise, miR-34a, miR-92a, miR-181c, and miR-210 are involved in regulating vascular endothelial function in diabetes mainly by inducing or reducing levels of oxidative stress markers [74,75,76,77,78].

Several studies have indicated that aberrant expression of the let-7 miRNA family (let-7b and let-7d), miR-149-5p, and miR-190a-5p regulate inflammatory pathways in diabetes-associated endothelial injury, and restoring expression of these miRNAs, similar to downregulation of miR-34a and miR-197 may contribute to decreased TNF-α, IL-6, IL-1β, MCP-1, VCAM-1, and ICAM-1 level [75,79,80,81,82]. It is worth noting that overexpression of miR-1 and miR-181c in hyperglycemic models led to reduced ET-1 and increased eNOS levels that improve endothelium-dependent vasodilatation [83,84].

Moreover, miR-26b-5p, miR-29b-3p, miR-31, miR-34a-5p, miR-93-5p, miR-181a-5p, miR-192-5p, miR-375-3p, and miR-425-5p have been demonstrated to induce EPC or EC apoptosis during sustained exposure to hyperglycemia, whereas miR-26a-5p and miR-29a exerted an opposite effect on EC viability [81,85,86,87,88,89]. The decreased expression of miR-9 and increased level of miR-134-5p are also considered to be possible links between diabetes mellitus and endothelial dysfunction [81,90]. Finally, miR-126, miR-132, miR-139-5p, miR-140-3p, miR-181a/b-5p, miR-221, and miR-342-3p have already been studied to be engaged in EC proliferation, migration, invasion, and tube formation, that presented them as the potential surrogate markers for angiogenesis upon hyperglycemic condition [91,92,93,94,95,96].

#### 3.1.2. MicroRNAs in Diabetes-Associated Endothelial to Mesenchymal Transition

Persistent activation of ECs under hyperglycemic conditions induces EndMT, which contributes to the initiation and progression of diabetes-accelerated atherosclerosis as a consequence [51]. EndMT is the process during which ECs lose their typical phenotypes, including cell-cell contact and cell polarity that results in a spindle-shaped morphology and the acquisition of mesenchymal-like or myofibroblastic phenotypes with gaining migratory and invasive properties [51,97,98]. Throughout EndMT, the expression of endothelial markers such as vascular endothelial cadherin (VE-cadherin), platelet endothelial cell adhesion molecule-1 (PECAM-1, also known as CD31), and von Willebrand Factor (vWF) decreases, whereas the expression of mesenchymal cell markers such as alpha-smooth muscle actin (α-SMA), smooth muscle protein 22 alpha (SM22α), vimentin, fibronectin, N-cadherin, calponin, and fibroblast specific protein-1 (FSP-1) increases [97,98]. EndMT may also lead to the delamination and migration of EC-derived mesenchymal cells into the underlying tissues and thus enhances the extracellular matrix (ECM) production [98]. Remarkably, the involvement of miRNAs in molecular mechanisms driving diabetic atherosclerosis-related EndMT still remains unclear.

Recent studies on culture cell models have revealed that upregulation of miR-142-3p and miR-448-3p alleviated diabetic vascular dysfunction by inhibiting EndMT [99,100]. The expression of the above-mentioned miRNAs is downregulated in diabetic atherosclerosis that promotes EndMT via activation of transforming growth factor β (TGF-β)/Smad signaling pathway, the most well-known inducer of EndMT [99,100]. High glucose-treated endothelial cells manifested a significant decrease in CD31, VE-cadherin, and Smad7, while α-SMA, vimentin, TGF-β1, phospho-Smad2, and phospho-Smad3 were increased [99,100]. In contrast, miR-328 is significantly upregulated in high glucose-induced EndMT, and its effects are related to triggering the mitogen-activated protein kinase kinases 1/2 (MEK1/2) and ERK1/2 phosphorylation [101]. Moreover, EndMT has been found to be abrogated by the transduction of antagomiR-328 [101]. These studies demonstrate the need for further research on the miRNA-EndMT regulatory network in diabetic atherosclerosis.

#### 3.1.3. MicroRNAs in Monocyte Differentiation/Macrophage Activation under Diabetic Condition

It is well documented that macrophage accumulation is a common feature of T2DM, and it is proposed as one of the principal mechanistic cores of diabetes-related atherosclerosis [19]. The recruitment of monocytes from the peripheral blood to the intima of the vessel wall and monocytes subsequent differentiation into macrophages, generating proinflammatory factors and activating other immune cells, promote atherosclerosis in the diabetic microenvironment [102]. In addition, the increased activation and migration of macrophages into different tissues, especially adipose tissue, are involved in the pathogenesis of chronic subclinical inflammation under hyperglycemic conditions [103]. According to the activation state and functions, macrophages can be divided into two heterogenous subsets: M1-type (classically activated macrophages) and M2-type (alternatively activated macrophages) [104,105]. M1 macrophages reveal proinflammatory phenotype and produce proinflammatory cytokines, such as TNF-α, IL-1β, IL-6, interleukin 12 (IL-12), and NO, whereas M2 macrophages are highly expressed in specific anti-inflammatory markers, such as interleukin 10 (IL-10), and argininase (Agr-1) [104,105,106,107].

Until now, only a few studies have explored the role of miRNAs in the context of monocyte differentiation and macrophage activation under a diabetic milieu. The overexpression of miR-99a and miR-448 has been found to prevent M1 phenotype activation and decrease the levels of inflammatory markers in T2DM [105,108]. In contrast, remarkably upregulated miR-130b, miR-330-5p, and miR-495 expression have been reported to enhance macrophage M1 polarization along with attenuating macrophage M2 polarization in the diabetic animal model [104,106,107]. Moreover, miR-483-3p has been observed to be overexpressed in M2-type macrophages in the aortic wall of patients with T2DM, that increased endothelial and macrophage apoptosis and impaired endothelial repair capacity [109]. Intriguingly, increased expression of miR-17-5p and miR-221 in diabetic monocyte-derived macrophages exert pro-proliferative effects on VSMCs by downregulation of cyclin-dependent kinase inhibitors, such as p21^Cip1^ and p27^Kip1^, resulting in the development of vascular dysfunction in T2DM [102].

Noteworthy, miRNAs are also involved in the regulation of lipid and lipoprotein metabolism, atherosclerotic plaque formation, as well as its size and stability [103,108,110,111,112]. T2DM is strictly related to atherogenic dyslipidemia, which is characterized by increased levels of triglycerides (TG), decreased high-density lipoprotein (HDL) cholesterol (C), and the existence of small dense low-density lipoprotein (LDL)-C [110]. miR-99a mimics have been shown to improve cholesterol metabolism, which was reflected by a significant decrease in the total cholesterol (TC), HDL-C, and LDL-C levels in the hyperglycemic environment [108]. Kimura et al. revealed that hyperinsulinemia might activate the sterol regulatory element-binding protein (SREBP)-1c/miR-33b pathway that consequently increased TG synthesis [110]. In addition, the elevated expression of miR-33b repressed the level of HDL-C and apolipoprotein A-1 (ApoA-1) by downregulation of the ATP-binding cassette, sub-family A, member 1 (ABCA1) protein which is engaged in the cholesterol efflux and is responsible for the generation of nascent HDL-C [110]. What is more, anti-miR-33 treatment has been observed to promote remodeling of diabetic plaque towards a more stable-appearing phenotype by decreasing macrophage content in the plaque [111]. Lipoprotein uptake by macrophages is believed to be one of the earliest pathogenic events in the nascent plaque and results in the formation of foam cells, a special form of lipid-laden macrophages [112]. miR-27b-3p, whose level is decreased in the diabetic model, may be involved in delaying the progression of atherosclerosis in hyperglycemic conditions as its overexpression reduces the uptake of oxidized low-density lipoprotein (ox-LDL) by macrophages [112]. Additionally, miR-145 overexpression increases apoptosis of monocytes, reduces their proliferation and macrophage infiltration, which attenuate inflammation and markedly reduce plaque size, partially by inhibition of NF-κB activation [103].

### 3.2. The Role of MicroRNAs in the Progression of T2DM-Associated Atherosclerosis

#### 3.2.1. MicroRNAs in Vascular Smooth Muscle Cell Proliferation and Migration under Diabetic Condition

Under physiological conditions, VSMCs are specialized to maintain a differentiated contractile phenotype which allows them to regulate vascular tone [113]. In response to arterial injury through hyperglycemic or inflammatory stimuli, VSMCs dedifferentiate and adopt a synthetic, proliferative, and migratory phenotype that is the most common pathological change in diabetic atherosclerosis [113]. Principal regulators in the maintenance of the mature VSMC phenotype include the transcription factor serum-response factor (SRF), SRF-associated coactivators such as myocardin, and TGF-β, whereas soluble factors such as platelet-derived growth factor (PDGF) promote dedifferentiation of VSMCs [113]. Moreover, the Krüppel-like factor (KLF) family of transcription factors, and in particular, KLF4, constitutes a key pluripotent transcription factor in VSMC differentiation and dedifferentiation via modulation of KLF4/myocardin/SRF axis [113]. Accumulating evidence has indicated that miRNAs play a pivotal role in the mechanisms determining VSMC phenotype in T2DM-related atherosclerosis.

The miRNA cluster containing miR-143 and miR-145, the most abundant VSMC miRNAs, influence VSMC plasticity in both human culture cells and those from T2DM patients [114,115,116,117]. Deficiency of miR-145 results in the increased migratory and proliferative capacity of VSMCs under high glucose conditions through targeting KLF4/myocardin pathway and Rho-associated coiled-coil forming protein kinase 1 (ROCK1), an important factor involved in atherosclerosis by increasing the permeability of ECs, enhancing the chemotaxis of macrophages, their transformation into foam cells, and VSMC phenotypic switching [114,115]. Interestingly, the divergent saphenous vein VSMC proliferation was observed between T2DM patients and non-diabetic ones undergoing coronary artery bypass grafting [116]. Overexpression of miR-143/-145 decreased VSMC proliferation in healthy individuals, whereas transfection of T2DM-VSMC with anti-miR-143/-145 increased cell proliferation, thus confirming the atheroprotective properties of these miRNAs [116]. Similarly, miR-217 and miR-132 exert their anti-proliferative and anti-migratory effects on VSMCs under high glucose conditions mimicking diabetes via suppression of ROCK1 and E2F transcription factor 5 (E2F5), respectively [118,119]. It should be noted that E2F5, a key member of the E2F family, controls the transcription of proliferation-related genes and the G1/S transition [119].

Another miRNA, which is one of the crucial regulators of high glucose-induced VSMC differentiation, is miR-24 [120,121,122,123]. It has been shown that high glucose-stimulated animal culture cells and/or carotid arteries in diabetic rats transfected with adenovirus-miR-24 precursor exhibited reduced VSMC proliferation, migration, and proinflammatory cytokine secretion, an effect that might be mediated by the inactivation of several pathways, including the HMGB1/NF-κB, PDGF-BB, and PI3K/Akt/mammalian target of rapamycin (mTOR) signaling pathways [120,121,122]. Moreover, miR-24 transfected into the same carotid artery in diabetic rats alleviated VSMC proliferation, and it was related to the regulation of the expression of Cyclin D1 and p21 through the inhibition of Wnt4/Dvl-1/β-catenin signaling pathway [123]. It has also been observed that miR-24 upregulation attenuated diabetic vascular remodeling [121,122,123,124,125].

As it was previously mentioned, the let-7 miRNA family has exerted not only anti-inflammatory effects in ECs but is also proposed as a master regulator of VSMC proliferation and differentiation [79]. In diabetes-associated atherosclerosis, the PDGF and TNF-α induced activation, increased proliferation, and migration of VSMCs are associated with decreased levels of let-7b and let-7d via Lin28b, a negative regulator of let-7 biogenesis [79]. Nevertheless, the restoration of let-7 to normal levels ensures an atheroprotective response [79].

In addition, several studies have proved that platelet-derived miR-223 suppresses the proliferation and dedifferentiation of VSMCs by decreasing platelet-derived growth factor receptor beta (PDGFRβ) and directly targeting the insulin-like growth factor-1 receptor (IGF-1R), which then activates the adenosine monophosphate-activated protein kinase (AMPK) phosphorylation [126,127]. However, miR-223 expression is pathologically reduced under diabetic conditions [126,127]. miR-9, miR-125a, and miR-322-5p have been identified as other miRNAs that contribute to the acquisition of a contractile phenotype of VSMCs [128,129,130]. It has been observed that decreased miR-9 expression in diabetic human VSMCs resulted in increased activity of KLF5, a positive regulator of VSMC dedifferentiation and transformation into synthetic phenotype, and a subsequent decrease in myocardin expression [128].

What is more, the reduced transfer of antiproliferative miR-126-3p from ECs to VSMCs has been observed under the hyperglycemic condition that leads to accelerated proliferation and migration of VSMCs [131]. Most recently, miR-126-3p has occurred to play a crucial role in T2DM VSMC metabolic memory through the activation of MAPK/ERK pathway, enhancing the efficiency of blockers of potassium channels Kv1.3 in VSMCs, thus preventing their proliferation, migration, and vessels remodeling [132].

The data on the relevance of miR-29c in the context of its atherogenic and antiatherogenic properties are inconsistent [133,134]. Torella et al. have revealed that overexpression of miR-29c with contemporaneous miR-204 inhibition upon hyperglycemia prevented exaggerated VSMC proliferation by regulation of epithelial membrane protein 2 (EMP2) and caveolin 1 (CAV1) as direct targets [133]. In contrast, other authors have suggested that hyperglycemia-induced upregulation of miR-29c via inhibition of KLF4 activity, thus stimulating VSMC proliferation [134].

Likewise, miR-19a, belonging to the miR-17-92 cluster, miR-138, and miR-504 promote a switch to the synthetic phenotype, and their expressions have been found to be increased in high glucose-induced culture conditions and diabetic animal models [135,136,137]. Moreover, another miRNA with a proven proatherogenic effect is miR-376b-3p, whose upregulation is observed in a diabetic state and it leads to the increased VSMC proliferation by suppression of KLF15, a negative regulator of proliferative processes [138]. In the given hyperglycemic condition, miR-221/-222 and miR-17-5p, similarly to miR-21-5p, have also been proposed to act upon VSMC proliferation and migration via downregulation of p27^Kip1^, a cyclin-dependent kinase inhibitor, and tropomyosin 1 (TPM1), a regulator of cytoskeletal actin filaments, respectively [102,139,140,141].

Importantly, T2DM is associated with not only hyperglycemia but also compensatory hyperinsulinemia in the setting of insulin resistance. miR-99a has been shown to be decreased in high-dose insulin environments that stimulated proliferation, migration, and dedifferentiation of VSMCs by attenuation of the inhibitory effects of miR-99a on IGF1-R and mTOR signaling pathways [142].

#### 3.2.2. MicroRNAs in Platelet Hyperactivity under Diabetic Condition

Platelets, although anucleate, are thought to be important cellular components involved in both the initiation and progression of atherosclerosis especially in the ensuing atherothrombotic sequelae [143]. T2DM modulates the function of platelets leading to their increased activation, aggregation, adhesiveness to ECs, and thrombus formation [144,145,146]. Various mechanisms have been proposed to be responsible for the diabetes-induced platelet hyperactivity, including altered calcium homeostasis with the overactivation of calpain, the calcium-activated cysteine protease, abnormal expression of surface glycoprotein receptors and adhesion molecules, and increased binding of fibrinogen [143]. It is worth mentioning that platelets are a rich source of miRNAs and functioning proteins (Dicer, TRBP2, and AGO2), which allow them to convert pre-miRNAs into mature miRNAs [147]. In addition, platelets release abundant miRNAs in the form of microvesicles [145,146]. Accordingly, platelet-specific miRNAs may facilitate the communication between platelets and other immune and vascular cells.

One of the most thoroughly studied miRNAs in relation to platelet hyperactivity under hyperglycemic condition are miR-223, together with miR-126, miR-140, and miR-26b, whose expressions are decreased in both platelets and megakaryocytes from T2DM patients [147]. Moreover, it occurred that altered platelet miR-223, and miR-26b, miR-140 expressions lead to upregulation of mRNA and protein levels of P2Y12 receptor and P-selectin (CD62), known as platelet hyperactivity marker [147]. Investigation of the role of miR-223 in platelet function under diabetic conditions revealed decreased Dicer activity, generating a lower amount of mature miR-223 [147]. Supporting these findings, other researchers observed that inhibition of calpain-dependent cleavage of Dicer normalizes miR-223 processing and restores platelet function [148]. On the other hand, AGO2 levels were comparable in healthy and diabetic platelets [148]. Furthermore, the expression of a subset of platelet-derived miRNAs, such as miR-142/-143 and miR-155, was reduced in human individuals and mice with T2DM [148]. Similar to miR-223, miR-126 may also play a role in the modification of the expression of the P2Y12 receptor in T2DM. Thus miR-126 mimics are able to reverse metallothionein 1 pseudogene 3-mediated upregulation of the P2Y12 receptor [149]. Of note, plasma levels of miR-126 were increased in T2DM and directly correlated with soluble P-selectin [150]. However, it occurred that the administration of aspirin in this group led to the subsequent decline in platelet reactivity and the decrease in miR-126 [150]. Therefore, de Boer et al. hypothesized that other platelet-enriched miRNAs, such as miR-16, miR-223, and miR-423, can be released from activated platelet into circulation in a similar fashion [150]. Recent studies have revealed that patients with T2DM also present significantly diminished expression of miR-30c originating from platelets, and its restoration to the desired level leads to downregulation of plasminogen activator inhibitor-1 (PAI-1) expression as a target, thereby prolonging the time to arterial thrombus formation [151].

Contrary to previous studies, Stratz et al. have demonstrated no major divergences in platelet miRNA profiles between the studied groups, neither T2DM and non-T2DM nor CAD and non-CAD [152]. Nevertheless, the authors have indicated that miR-377-5p, miR-628-3p, and miR-3137 may serve as relatively stable predictors of group membership [152].

#### 3.2.3. MicroRNAs in Diabetes-Associated Calcification

Vascular calcification is an active process strongly associated with atherosclerotic plaque evolution and results in an increased incidence of cardiovascular events and mortality [153,154]. It refers to the pathological deposition of calcium and phosphate minerals in the inner or middle layer of the vascular wall [154].

So far, data concerning the role of miRNAs in the pathogenesis of vascular calcification in T2DM are limited. It has been observed that miR-204 is downregulated in asymptomatic diabetic patients with higher coronary artery calcification (CAC) scores assigned in coronary computed tomography [154]. What is interesting, the expression of miR-204 is negatively correlated with glycated hemoglobin A1c (HbA1c), and it has occurred to be a significant and independent risk factor for the presence of CAC [154]. Similarly, miR-95-5p, miR-6776-5p, miR-3620-5p, and miR-4747-5p are suppressed in high glucose-induced calcification of VSMCs [155]. Nonetheless, the exact mechanisms of the aforementioned miRNAs remain largely uncharacterized [154,155]. The overexpression of miR-34c-5p, but not miR-34c-3p, may alleviate VSMC calcification under hyperglycemic conditions [153]. In turn, miR-128-3p accelerates cardiovascular calcification and insulin resistance in the T2DM animal experimental model through the activation of the Wnt signaling pathway, which has been proved to be involved in the formation of calcium phosphate deposits in the vessels [156].

**Figure 2:**
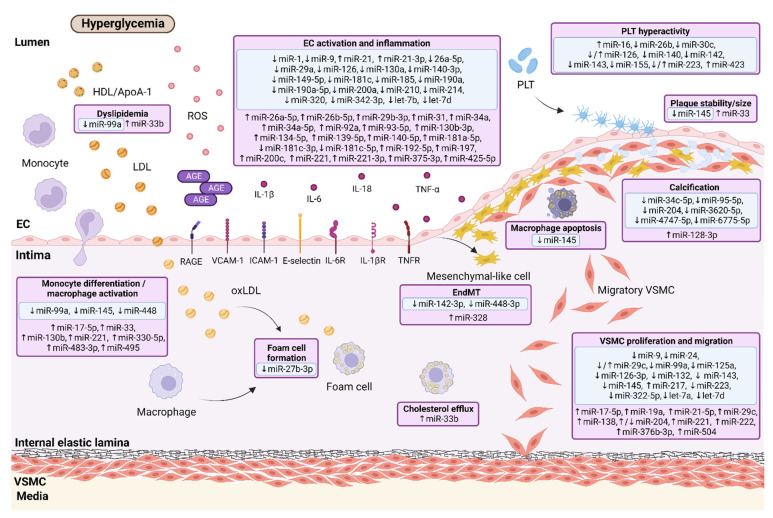
MicroRNAs implicated in T2DM-related atherosclerosis. Positive/atheroprotective (in green frame) and negative/atherogenic (in violet frame) effects of miRNAs on every step of the atherosclerotic process under diabetic conditions are shown. Chronic hyperglycemia induces inflammation, and oxidative stress leading to the cascade of processes, including EC activation, migration of monocytes into the intima and their maturation into macrophages, foam cell formation by oxLDL uptake, PLT hyperactivity, VSMC proliferation and migration into the intima. Single miRNAs are also found to be engaged in EndMT, calcification, and regulation of plaque size/stability. miR—microRNA; EC—endothelial cell; EndMT—endothelial-to-mesenchymal transition; VSMC—vascular smooth muscle cell; PLT—platelet; ROS—reactive oxygen species; IL—interleukin; TNF-α—tumor necrosis factor α; IL-6R—interleukin 6 receptor; IL-1βR—interleukin 1β receptor; TNFR—tumor necrosis factor receptor; AGE—advanced glycation end-product; RAGE—receptor for advanced glycation end-products; VCAM-1—vascular cell adhesion molecule-1; ICAM-1—intercellular adhesion molecule-1; HDL—high-density lipoprotein; LDL—low density lipoprotein; ApoA-1—apolipoprotein A-1; ox-LDL—oxidized low-density lipoprotein. ↑ upregulation; ↓ downregulation. Created with BioRender.com.

The involvement of various miRNAs in particular stages of the initiation and progression of T2DM-induced atherosclerosis is presented in Figure 2.

## 4. Clinical Research on Circulating MicroRNAs in T2DM and CAD

Circulating miRNAs were first detected in the samples of human serum and plasma in 2008, and subsequently, they have been found in a variety of body fluids, such as saliva, urine, breast milk, colostrum, bronchial lavage, cerebrospinal fluid, peritoneal fluid, pleural fluid, amniotic fluid, seminal fluid, ovarian follicular fluid, and even tears [28,157,158,159]. In the whole blood, miRNAs are also discovered in peripheral blood mononuclear cells (PBMCs) such as lymphocytes, monocytes, and macrophages, which are suspended in plasma and constitute an additional source of miRNAs [160]. Contrary to proteins and different RNA classes, extracellular miRNAs are highly stable in circulation and resistant not only to enzymatic degradation by RNase but also to deleterious conditions, including boiling, repeated freeze-thaw cycles, and pH fluctuation [28]. Consequently, serum or plasma specimens can be stored at room temperature for up to four days and at −20 °C or −80 °C for several months without remarkable degradation of miRNAs [161]. Moreover, miRNA sequences are conserved among species, and their detection is based on modern, minimally invasive technologies, which ensure high testing sensitivity and specificity [162,163]. miRNAs could be easily and repetitively detectable using reverse transcription quantitative real-time polymerase chain reaction (RT-qPCR), different types of microarrays, and alternative techniques, including northern blotting, next-generation sequencing (NGS) or NanoString nCounter [163,164]. Finally, miRNAs are tissue-specific, which allows the identification of their origin and establishing a disease-specific expression pattern of miRNAs [165]. Interestingly, miRNA signatures seem to be more robust biomarkers than single miRNAs and are more likely to adequately reflect the complexity of disease pathophysiology [165].

The listed features of miRNAs suggest that they are promising as ideal candidates for disease biomarkers. Findings from preclinical and basic research studies highlight that miRNAs may serve as a fingerprint for T2DM-induced atherosclerosis. Therefore, the clinical usefulness of circulating miRNAs as early diagnostic, prognostic, and predictive biomarkers for T2DM, CAD, and CAD related to T2DM have been discussed in detail.

### 4.1. MicroRNAs as Potential Biomarkers for T2DM

The pathogenesis of T2DM arises from the interplay of genetic, epigenetic, and environmental factors, which may impair insulin sensitivity in target tissues, including mainly the liver, adipose tissue, skeletal muscles, and insulin secretion from pancreatic beta cells [8]. It is commonly known that the compensatory hyperinsulinemia to maintain euglycemia in the setting of peripheral insulin resistance ultimately leads to the deterioration of beta cells, their exhaustion, and eventual death [8]. The state of relative insulin deficiency in chronically elevated blood glucose levels consequently generates the onset of T2DM [8]. Despite the progress made in the exploration of mechanisms underlying the pathogenesis of T2DM, understanding this complex metabolic disorder at the molecular level and searching for miRNA-based biomarkers used for the early detection and identification of highly susceptible individuals seems to be relevant to avoid vascular complications and improve patient quality of life by providing appropriate management.

A growing number of reports have revealed T2DM-related expression changes of circulating miRNAs. However, only seventeen studies have attempted to perform miRNA expression profiling to find a unique miRNA pattern [166,167,168,169,170,171,172,173,174,175,176,177,178,179,180,181,182], while the remaining researchers have focused on miRNA selection based on the review of available scientific literature or analysis of miRNA databases. Therefore, it seems that the concept of initial miRNA screening with their further validation, especially in previously unexplored ethnic groups, holds much potential for the discovery of novel miRNAs associated with T2DM and to select of those molecules that have the greatest discriminatory power.

The pioneering study that established a plasma signature of 13 miRNAs in T2DM individuals based on microarray profiling was the prospective population-based study conducted by Zampetaki et al. [174]. The researchers revealed lower expression of miR-15a, miR-20b, miR-21, miR-24, miR-29b, miR-126, miR-150, miR-191, miR-197, miR-223, miR-320, miR-486, and a modest increase in miR-28-3p expression in diabetic patients compared to controls [174]. Interestingly, it has occurred that 70% of T2DM cases and 92% of the control group were correctly classified using the five most significant miRNAs, including miR-15a, miR-28-3p, miR-126, miR-223, and miR-320 [174]. Moreover, this miRNA panel allowed the prediction of T2DM in normoglycemic patients because their expression was disturbed about 10 years prior to disease diagnosis, confirming the value of miRNAs as potential predictive and diagnostic biomarkers [174]. Similarly, Karolina et al. have identified a set of circulating miRNAs that displayed altered expression in diabetes, but they have observed contrasting expressions of miR-150 and miR-320 in T2DM cohorts, probably due to a distinct sample type than in previous report (plasma vs. whole blood) [172,180]. What is more, among seven diabetes-related serum miRNAs, apparently upregulated miR-34a showed the highest standardized canonical discriminant function coefficients allowing correct judgment of 70.6% of T2DM subjects [183]. Evidence for the clinical usefulness of these miRNA panels was provided by the discovery of miRNA-regulated pathways, including insulin production (miR-30d), insulin secretion (miR-9, miR-124a, miR-375), insulin signaling and action in target tissues (miR-27a, miR-29a, miR-144, miR-146a, miR-150, miR-182, miR-192, miR-320), especially translocation of glucose transporter-4 (GLUT4) receptor from the intracellular storage to the plasma membrane of the insulin-responsive cells to allow uptake of glucose (miR-150) [172,180,183]. Additionally, other miRNAs considered as T2DM biomarkers have been found to be implicated in insulin signaling pathways (let-7f-5p, miR-24-3p, miR-145-5p, miR-214-3p), beta cell function, and insulin secretion (let-7b-5p, miR-375, miR-720, miR-770-5p), insulin resistance (miR-30d, miR-145-5p, miR-199a, miR-330), and glucose or lipid metabolism networks (miR-29, miR-33a, miR-122, miR-155) [175,184,185,186,187,188,189,190,191,192,193].

Recent clinical data have indicated a close link between specific miRNAs and inflammation in T2DM [194]. As observed, miR-126 and miR-146a are among the most commonly reported anti-inflammatory miRNAs with the potential to be used as epigenetic biomarkers for T2DM [195,196,197,198,199,200,201]. It has occurred that normoglycemic individuals with decreased expression of plasma miR-126 with a cut-off point of less than 35 are more likely to develop T2DM in the next two years [197]. Interestingly, the introduction of miR-126 to the established conventional model, including age, gender, body mass index (BMI), blood glucose, and HbA1c, allowed to obtain higher discriminatory power for T2DM patients with an area under the curve (AUC) of 0.893 than for miR-126 or the model alone (0.792 vs. 0.826) [196]. In line with receiver operating characteristic (ROC) analysis results, downregulated expression of endothelial-derived miR-126 in whole blood is suitable for differentiating T2DM subjects from healthy controls with an excellent AUC of 0.932 [198]. On the other hand, Weale et al. have pointed out that increased expression of miR-126, also measured in peripheral blood, achieved a satisfactory overall predictive ability in diagnosing T2DM, although this value was remarkably lower than that calculated for HbA1c commonly recognized as one of the diagnostic indices for diabetes (AUC of 0.646 vs. 0.861) [199]. It is worth mentioning that upregulation of another inflammation-related miRNA, miR-146a, demonstrated a good discriminatory power for T2DM screening with an AUC of 0.725 and significantly increased the risk for new T2DM (crude odds ratio; OR, 4.333; 95% CI, 1.935–9.705; *p* < 0.001) in patients belonging to the group of the highest miR-146a tertile levels (≥1.339) [195]. Similar to miR-126, adding miR-146a as an auxiliary component of the established conventional model comprising age, sex, BMI, smoking, alcohol drinking, hypertension, family history of diabetes, TG, HDL-C, LDL-C, TC, is able to enhance AUC value from 0.753 to 0.844 [195]. In addition to their potential diagnostic values, the profile of circulating miRNAs could also provide precious information about the pathophysiology of the disease. It merits special mention that both miR-126 and miR-146a are closely linked to generally approved inflammatory and endothelial dysfunction markers, including IL-6, IL-8, TNF-α, NF-κB, VCAM-1 or interleukin 1 receptor-associated kinase 1 (*IRAK1*), tumor necrosis factor receptor-associated factor 6 (*TRAF6*) as well as they can lead to the accumulation of dysfunctional endothelial senescent cells and the shift of circulating angiogenic cells from a proangiogenic to a proinflammatory profile [194,202,203,204,205,206,207,208]. Furthermore, these miRNAs have been found to be associated with increased platelet activation, oxidative stress, endoplasmic reticulum stress or apoptosis (caspase-3) indices, and decreased plasma antioxidant capacity [147,204,205]. Aside from the above, miRNAs such as miR-18a, miR-34c, miR-21-5p, miR-103b, miR-122, miR-155, miR-181b, miR-574-3p, and miR-576-3p may act as mediators of inflammatory response in patients with T2DM, although some authors have suggested that miR-34c-5p, miR-146a, miR-155, miR-574-3p, and miR-576-3p did not correlate with the level of proinflammatory cytokines, except for the poor association of miR-574-3p with MCP-1 [181,194,203,206,207,209,210,211,212,213].

Considering that obesity is an established risk factor for T2DM, and adipose tissue is a highly active metabolic and endocrine organ releasing an array of hormones and cytokines (adipocytokines), identifying obesity-related miRNAs as candidate biomarkers for T2DM seems to be essential [8]. So far, circulating miR-130a, miR-326, and miR-3666 have been recognized to modulate the adiponectin pathway [170,182,214]. Notably, upregulated miR-326 is an independent predictor of plasma adiponectin levels, irrespective of age, sex, and BMI [170]. Moreover, significantly altered expression of miR-130a and miR-3666 inhibits adiponectin gene expression, indirectly affecting adipocyte differentiation and reducing insulin sensitivity, promoting apoptosis of pancreatic beta cells, and diminishing their differentiation [182,214]. The observed positive correlations of miR-326 and miR-3666 with HbA1c and/or blood glucose levels are clinically relevant as they can also be used to monitor glycemic control [182,214]. Likewise, other dysregulated miRNAs in T2DM subjects, including pancreatic-islet specific miR-7, miR-21 and miR-148a, miR-217, miR-221, miR-222 displayed positive association with markers of glycemic control, whereas miR-146a, miR-185, miR-222-3p, and miR-342-3p showed the negative one [204,206,208,213,215,216,217,218,219,220].

As presented in Table 1, miR-15a, miR-23a, miR-30c, miR-103a/103b, miR-126, miR-210, miR-320b, miR-499, miR-572, and miR-766-3p have obtained the highest capability to distinguish T2DM from normal glucose tolerant patients with an AUC value above 0.8 and simultaneously sensitivity, specificity reaching even 100% [171,179,197,198,200,221,222,223,224,225,226], while the other miRNAs regarded as T2DM biomarkers have shown the satisfactory or good discriminatory power [171,179,192,195,196,199,218,227,228,229,230]. Therefore, it is proposed that rather than focusing on single miRNAs that are involved in T2DM development, an integrated view should also take into account the combination of several miRNAs whose diagnostic ability, sensitivity, and specificity may be even stronger [166,169,170,173,212,216,231].

The results obtained in the determination of miRNA from various biological fluids remain controversial, which is why special attention should be paid to the sample type used in the study when miRNAs are considered as T2DM biomarkers. It has been demonstrated that expression of miR-126 and miR-342 in different blood compartments such as serum, plasma, or whole blood can be both upregulated or downregulated [166,169,194,196,199,232,233]. Similarly, miR-34a has presented an opposite pattern of expression between samples, being increased in plasma or PBMCs, and decreased in whole blood [234,235,236]. However, miR-15a and miR-223 have revealed the same expression profile regardless of the biological fluid used (whole blood, plasma vs. PBMC, plasma, platelets), except for the inverse miR-223 expression in serum [147,148,174,222,237,238,239]. Interestingly, Monfared et al. have investigated the expression of miR-126 and miR-135a in saliva for the first time, suggesting that it is an equally promising non-invasive research material [200]. It should also be noted that miRNA profile may be affected by sex- and ethnicity-associated differences, although data in this field are scarce and require further elucidation [240,241].

Additionally, it is emphasized that the expression of miRNAs may be disparate between the same treatment-naïve T2DM individuals and those during anti-diabetic therapy, but the group of anti-diabetic drugs had no effect on the expression profile of the assessed miRNAs [232,242]. Several studies evaluating miRNAs as candidate biomarkers for T2DM have also noticed the possible usefulness of selected miRNAs for monitoring therapeutic responses in patients with diabetes [169,170,196].

The studies showing the potential clinical utility of miRNAs as biomarkers for T2DM are summarized in Table 1.

**Table 1:** Circulating microRNAs as potential biomarkers for type 2 diabetes mellitus.

miRNA	Expression Change	Sample Type	Assay Method	Number of Samples	Ethnicity	Age [Years]	Gender (Male/Female, *n*)	BMI [kg/m^2^]	Duration of T2DM [Years]	HbA1c [%]	Value of Biomarker (AUC; 95% CI; SV [%]; SP [%])	Author, Year (Reference)
let-7b-5p	Up	Serum	RT-qPCR	T2DM (29) HC (25)	Emirati	55.6 ± 9.0 42.8 ± 12.7	13/16 9/16	31.5 ± 6.0 28.3 ± 6.5	Newly diagnosed	7.6 ± 1.6 5.2 ± 0.4	N/A	Aljaibeji et al., 2022 [185]
miR-766-3p	Down	Serum	qPCR	T2DM (108) HC (68)	Chinese	46.80 ± 18.43 46.56 ± 16.85	62/46 40/28	25.61 ± 6.59 24.14 ± 4.54	Newly diagnosed	9.60 ± 2.48 5.57 ± 0.81	0.880 88.9; 75.0	Cao et al., 2022 [221]
miR-33a, miR-122	Up	Whole blood	RT-qPCR	T2DM (50) HC (50)	Iranian	55.9 ± 8.9 47.4 ± 9.2	Only male	27.0 ± 3.4 25.0 ± 3.3	Diagnosed	N/A	N/A	Masoudi et al., 2022 [193]
miR-499	Down	Serum	RT-qPCR	T2DM (60) HC (60)	Egyptian	Age-matched	Sex-matched	BMI-matched	N/A	N/D	0.970 90.0; 96.6	Oraby et al., 2022 [226]
miR-145-5p	Down	Plasma	RT-qPCR	T2DM (20) HC (20)	Iranian	57.05 ± 1.99 51.07 ± 2.29	N/A	28.25 ± 0.95 26.94 ± 0.08	Diagnosed	8.15 ± 0.4 5.29 ± 0.06	0.77 (0.60–0.93)	Shahrokhi et al., 2022 [192]
miR-107	Up	Serum	RT-qPCR	T2DM (53) HC (54)	Lithuanian	65 (44–83) 62 (48–80)	24/29 25/29	34.14 ± 5.92 28.07 ± 5.25	15 (5–30) –	8.23 ± 2.14 5.46 ± 0.49	N/A	Šimonienė et al., 2022 [242]
miR-21	Up	Plasma	RT-qPCR	T2DM (24) HC (29)	Iranian	54.42 ± 7.76 50.42 ± 6.14	15/9 19/10	N/A	Newly diagnosed	7.16 ± 0.16 5.15 ± 0.54	0.78 (0.64–0.92) 79.17; 81.48	Yazdanpanah et al., 2022 [227]
miR-720	Up	Whole blood	RT-qPCR	T2DM (50) HC (50)	Chinese	57 ± 8.2 55 ± 7.8	24/26 27/23	26.2 ± 4.1 23.1 ± 3.8	Newly diagnosed	9.89 ± 2.74 3.21 ± 1.27	N/A	Lu et al., 2021 [191]
miR-135a	Up	Saliva	RT-qPCR	T2DM (40) HC (40)	Iranian	47 ± 1.6 46 ± 1.4	26/54 ^1^	27.6 ± 1.3 26.4 ± 1.9	Diagnosed	7.6 ± 0.3 4.0 ± 0.2	0.007 95.0; 95.0	Monfared et al., 2021 [200]
miR-126	Down										1 100.0; 100.0	
miR-33a-5p	Up	Plasma	RT-qPCR	T2DM (20) HC (20)	Iranian	57.05 ± 1.99 51.07 ± 2.29	10/10 10/10	28.25 ± 0.95 26.94 ± 0.08	Diagnosed	8.15 ± 0.4 5.29 ± 0.06	0.71 (0.542–0.889)	Saeidi et al., 2021 [230]
miR-7-1-5p	Down										NS	
miR-770-5p	Up	Serum	RT-qPCR	T2DM (20) HC (20)	Chinese	32–61 29–64	14/8 14/8	N/A	Newly diagnosed	N/A	N/A	Wang et al., 2021 [190]
miR-30a-5p, miR-126-3p, miR-182-5p, miR-1299	Up	Whole blood	RT-qPCR	T2DM (92) HC (974)	South African	58.15 ± 10.62 45.22 ± 15.3	19/73 286/688	31.5 ± 8.0 27.4 ± 7.8	Newly diagnosed	7.3 ± 1.9 5.6 ± 0.5	N/A	Weale et al., 2021 [232]
miR-30a-5p, miR-30e-3p, miR-126-3p, miR-182-5p, miR-1299				T2DM (188) HC (974)		57.88 ± 11.97 45.22 ± 15.3	37/151 286/688	30.7 ± 6.4 27.4 ± 7.8	Diagnosed	8.9 ± 2.4 5.6 ± 0.5		
miR-126-3p	Up	Whole blood	RT-qPCR	T2DM (94) HC (972)	South African	58.4 ± 10.6 45.2 ± 15.3	19/75 284/688	31.3 ± 8.0 27.4 ± 7.9	Newly diagnosed	7.4 5.6	0.646 (0.576–0.717) 55.6; 70.8	Weale et al., 2021 [199]
miR-122	Up	Whole blood	RT-qPCR	T2DM (30) HC (30)	Iranian	53.03 ± 9.66 55.37 ± 8.47	15/15 15/15	30.27 ± 3.11 29.80 ± 2.89	Diagnosed	7.29 ± 1.22 4.54 ± 0.20	N/A	Zeinali et al., 2021 [194]
miR-126-3p, miR-146a	Down											
miR-29, miR-155	Up	Serum	qPCR	T2DM (59) HC (72)	Xinjiang Uygurian	48.45 ± 7.36 44.56 ± 3.58	27/32 36/36	28.50 ± 4.69 21.94 ± 1.33	Diagnosed	N/A	N/A	Zhu et al., 2021 [189]
miR-330	Up	Serum	RT-qPCR	T2DM (100) HC (100)	Indian	> 50 (40.0%) > 50 (50.0%)	57/43 55/45	> 25 (34.0%) > 25 (15.0%)	Newly diagnosed	N/A	N/A	Ali Beg et al., 2020 [188]
let-7f-5p, miR-24-3p, miR-214-3p	Down	Whole blood	miSript miRNA PCR array, RT-qPCR	T2DM (40) HC (16)	Greek	59 (35–75) 45 (19–52)	19/21 7/9	29.3 (21.5–46.5) 24 (21.3–24.0)	5 (0–26) –	6.7 (5.2–12.1) –	N/A	Avgeris et al., 2020 [175]
miR-34a	Up	Plasma	RT-qPCR	T2DM (30) HC (30)	Indian	38.9 ± 5.8 40.6 ± 5.95	19/11 17/13	27.8 ± 6.29 23.33 ± 3.57	4.55 ± 4.3 –	7.51 ± 1.22 4.89 ± 0.29	N/A	Banerjee et al., 2020 [235]
miR-126-5p, miR-181b	Down	Whole blood	RT-qPCR	T2DM (30) HC (30)	Iranian	55.4 ± 5.3 53.5 ± 7.2	14/16 16/14	N/A	Newly diagnosed	8.62 ± 1.74 5.1 ± 0.24	N/A	Dehghani et al., 2020 [207]
miR-103a	Up	Plasma	RT-qPCR	T2DM (48) HC (50)	Han Chinese	52.6 ± 9.13 45.62 ± 8.58	26/22 26/24	25.52 ± 2.89 24.56 ± 3.40	Newly diagnosed	9.16 ± 1.05 5.15 ± 0.32	0.998 (0.993–1.0) 97.9; 98.0	Luo et al., 2020 [225]
miR-103b	Down										0.964 (0.920–1.0) 98.0; 91.7	
miR-135	Up	Plasma	RT-qPCR	T2DM (40) HC (40)	Iranian	53.69 ± 5.69 33.59 ± 7.58	N/A	30.11 ± 1.01 25.23 ± 2.43	Newly diagnosed	7.63 ± 0.41 4.70 ± 2.30	N/A 50.5; 91.2	Monfared et al., 2020 [229]
miR-222	Up	Plasma	RT-qPCR	T2DM (30) HC (30)	Iranian	52.42 ± 8.77 51.44 ± 6.04	20/10 21/9	28.17 ± 5.46 27.60 ± 3.9	Newly diagnosed	7.34 ± 1.08 5.76 ± 0.41	N/A	Sadeghzadeh et al., 2020 [239]
miR-15a	Down											
miR-19a, miR-130a, miR-148b, miR-223	Up	Serum	RT-qPCR	T2DM (102) HC (68)	Mongolian (Chinese)	N/A	N/A	N/A	Newly diagnosed	N/A	N/A	Yan et al., 2020 [238]
let-7b-5p, miR-1-3p, miR-24-3p, miR-34a-5p, miR-98-5p, 133a-3p	Down	Whole blood	qPCR	T2DM (40) HC (37)	Greek	59 (35–75) 49 (19–69)	19/21 19/18	29.3 (21.5–46.5) 26.9 (21.3–36.3)	5 (0–26) –	6.7 (5.2–12.1) 5.6 (5.0–6.1)	N/A	Kokkinopoulou et al., 2019 [234]
miR-21 ^2^	Up	Plasma	RT-qPCR	T2DM (27) HC (44)	Italian	61.69 ± 7.59 59.3 ± 9.82	10/17 15/29	29.26 ± 5.83 25.11 ± 3.32	Newly diagnosed	6.64 ± 0.6 5.80 ± 0.38	0.699 93.0; 35.0	La Sala et al., 2019 [228]
miR-30c	Down	Plasma	qPCR	T2DM (47) HC (32)	Han Chinese	60.5 ± 11.1 58.6 ± 8.1	23/24 17/15	24.76 ± 3.29 24.49 ± 2.30	Newly diagnosed	9.15 ± 1.02 5.36 ± 0.35	0.916 (0.853–0.980) 87.9; 87.2	Luo et al., 2019 [224]
miR-486-3p	Up	Plasma	RT-qPCR	T2DM (29) HC (30)	Israeli Arab/Jewish	64 ± 10 31 ± 11	18/11 15/15	30 ± 5 25 ± 4	Newly diagnosed	7.7 ± 1.9 5.1 ± 0.3	N/A	Meerson et al., 2019 [240]
miR-423	Down											
miR-342	Up	Serum	RT-qPCR	T2DM (50) HC (50)	Egyptian	62.06 ± 1.26 62.22 ± 0.69	Only female	27.58 ± 0.28 23.82 ± 0.14	12.06 ± 0.30 –	10.75 ± 0.17 4.10 ± 0.68	N/A	Seleem et al., 2019 [233]
miR-450	Down											
miR-3666	Up	Serum	qPCR	T2DM (60) HC (30)	Chinese	45.81 ± 5.92 N/A	36/24 N/A	25.12 ± 0.31 N/A	Diagnosed	N/A	N/A	Tan et al., 2019 [214]
miR-146a	Down	Plasma, PBMC	RT-qPCR	T2DM (30) HC (30)	Iranian	57 (48–61) 50.5 (45.75–61)	11/19 9/21	27.13 ± 4.15 27.22 ± 3.26	8.53 ± 1.29 –	7.4 (6.7–8) 5.1 (5–5.4)	N/A	Alipoor et al., 2018 [206]
miR-9, miR-375	Up	Whole blood	RT-qPCR	T2DM (30) HC (30)	Bahrainis	60 ± 12 56 ± 5.1	12/18 14/16	25.7 ± 5.2 24.2 ± 4.6	15 ± 4.4 –	8.68 ± 2.6 5.03 ± 0.7	0.783 (0.665–0.902) ^3^	Al-Muhtaresh et al., 2018 [231]
miR-210	Up	Plasma	RT-qPCR	T2DM (54) HC (20)	Egyptian	56.5 ± 7.7 58.1 ± 1.1	29/25 11/9	30.7 ± 5.3 23.2 ± 0.2	10.8 ± 7.8 –	8.3 ± 1.1 4.8 ± 0.4	0.950 87.0; 100.0	Amr et al., 2018 [223]
miR-126	Down										0.960 96.3; 95.0	
let-7b ^3^, miR-29a, miR-144 ^3^	Up	Plasma	Microarray, RT-qPCR	T2DM (112) HC (94)	Han Chinese	54.75 ± 7.53 52.84 ± 8.85	69/43 53/41	27.11 ± 3.17 23.86 ± 3.27	Newly diagnosed	7.58 ± 1.54 5.16 ± 0.39	0.871 (0.822–0.919) ^3^ 79.5; 81.9	Liang et al., 2018 [173]
miR-142 ^3^	Down											
let-7e-5p, let-7f-5p, miR-15b-5p, miR-99b-5p, miR-103a-3p	Up	Whole blood	sRNA-Seq, RT-qPCR	T2DM (12) HC (12)	South African	54.8 ± 7.5 52.1 ± 7.8	Only female	33.5 ± 8.9 27.3 ± 5.8	Newly diagnosed	N/A	N/A	Matsha et al., 2018 [177]
miR-30d	Up	Plasma	RT-qPCR	T2DM (30) HC (30)	Asian Indian	50.5 ± 6.3 42.1 ± 7.8	21/9 21/9	27.3 ± 4.6 27.3 ± 4.7	3.10 ± 0.99 –	8.4 ± 2.0 5.5 ± 0.4	N/A	Sucharita et al., 2018 [187]
miR-126	Down	Whole blood	RT-qPCR	T2DM (45) HC (45)	Bahrainis	61 ± 12 53 ± 8.6	23/22 21/24	25.4 ± 4.8 24 ± 4.5	16 ± 6 –	7.4 ± 8.3 3.64 ± 1.1	0.932 (0.858–1.000)	Al-Kafaji et al., 2017 [198]
miR-148a-3p	Up	Plasma	RT-qPCR	T2DM (9) HC (9)	Italian	60.2 ± 8.0 57.9 ± 8.9	2/7 4/5	29.6 ± 7.8 23.7 ± 3.3	Newly diagnosed	6.4 ± 2.7 5.5 ± 2.4	N/A	de Candia et al., 2017 [220]
miR-222-3p, miR-342-3p	Down											
miR-26b, miR-126, miR-140, miR-223	Down	Plasma, Platelet	RT-qPCR	T2DM (28) HC (23)	Hungarian	53 (50–59) 53 (34–60)	15/13 12/11	32.9 (30.3–40.2) 24 (22.1–25.9)	10 (8.0–14.5) –	7.5 (7.0–8.8) –	N/A	Fejes et al., 2017 [147]
miR-126-3p	Down	Plasma (MPs)	RT-qPCR	T2DM (68) HC (53)	Italian	60 ± 1 57 ± 1	42/26 30/23	30 ± 1.6 25 ± 0.4	>5 –	N/A	N/A	Giannella et al., 2017 [205]
miR-223-3p	Down	PBMC	RT-qPCR	T2DM (16) HC (18)	Han Chinese	57 ± 9 53 ± 11	8/8 12/6	N/A	Newly diagnosed	N/A	N/A	Long et al., 2017 [237]
miR-217	Up	Serum	qPCR	T2DM (186) HC (195)	Chinese	54.87 ± 11.65 54.12 ± 9.45	95/91 99/96	25.30 ± 3.11 25.10 ± 3.27	6.39 ± 6.31 –	8.10 ± 2.09 5.36 ± 0.32	N/A	Shao et al., 2017 [219]
miR-34a, miR-125b	Up	PBMC	RT-qPCR	T2DM (73) HC (52)	Chinese	56.81 ± 11.85 Age-matched	38/35 Sex-matched	N/A	4.54 ± 5.41 –	8.50 ± 2.09 5.82 ± 1.07	N/A	Shen et al., 2017 [236]
miR-7	Up	Serum	RT-qPCR	T2DM (76) HC (74)	Chinese	48.5 ± 14.5 48.8 ± 15.2	50/26 41/33	25.2 ± 3.7 23.0 ± 2.7	1.8 ± 2.6 –	9.9 ± 2.9 5.3 ± 0.4	0.76 (0.68–0.83)	Wan et al., 2017 [218]
		Serum (exosome-free)									0.75 (0.67–0.83)	
miR-18a	Up	PBMC	RT-qPCR	T2DM (117) HC (105)	Chinese	51.68 ± 8.77 49.26 ± 9.09	68/49 58/47	27.44 ± 3.08 24.18 ± 2.86	Newly diagnosed	7.51 ± 1.42 5.19 ± 0.42	0.851 (0.800–0.902) ^3^ 78.6; 80.0	Wang et al., 2017 [212]
miR-34c	Down											
miR-96-5p, miR-144-3p, miR-454-3p, miR-455-5p	Up	Serum	miRNA qPCR array, RT-qPCR	T2DM (10) HC (5)	Chinese	58.2 ± 7.7 56.4 ± 3.7	4/6 2/3	N/A	Newly diagnosed	N/A	N/A	Yang et al., 2017 [176]
miR-409-3p, miR-665, miR-766-3p	Down											
miR-574-3p	Down	Serum	RT-qPCR	T2DM (64) HC (44)	Ecuadorian	61 (37–85) 53 (32–87)	24/40 13/31	29.5 (22–49) 28.7 (23–42)	Diagnosed	7.0 (3.2–12.5) 5.6 (3.9–6.9)	N/A	Baldeón et al., 2016 [211]
miR-451a, miR-4534	Up	Serum	Microarray, RT-qPCR	T2DM (154) HC (69)	Chinese	61.1 ± 12.4 54.2 ± 10.7	70/84 30/39	N/A	Newly diagnosed	N/A	N/A	Ding et al., 2016 [178]
miR-320d, miR-572, miR-3960	Down											
miR-221, miR-222	Up	Serum	RT-qPCR	T2DM (30) HC (20)	Chinese	60.79 ± 11.11 59.78 ± 11.23	Only female	28.88 ± 1.18 20.12 ± 1.69	Newly diagnosed	7.60 ± 0.33 4.56 ± 0.45	N/A	Li et al., 2016 [217]
miR-30c	Down	Plasma, Platelet	RT-qPCR	T2DM (40) HC (50)	Han Chinese	58.6 ± 6.2 52.2 ± 5.5	21/29 31/19	27.3 ± 4.2 23.6 ± 2.8	Diagnosed	7.3 ± 0.5 5.3 ± 0.2	N/A	Luo et al., 2016 [151]
miR-21, miR-30d ^3^, miR-34a ^3^, miR-148a	Up	Plasma	RT-qPCR	T2DM (31) HC (27)	American	52.9 ± 2.0 25.3 ± 2.2	15/16 15/12	34.1 ± 1.3 24.1 ± 0.9	Diagnosed	6.56 ± 0.11 5.24 ± 0.06	0.928 ^3^ 90.32; 88.89	Seyhan et al., 2016 [216]
miR-571, miR-661, miR-770-5p, miR-892b, miR-1303 ^4^	Up	Serum	TLDA, RT-qPCR	T2DM (92) HC (92)	Chinese	47.7 ± 13.9 50.2 ± 14.2	58/34 56/36	25.6 ± 4.5 23.6 ± 2.0	2.1 ± 2.7 –	9.8 ± 2.9 5.3 ± 0.4	0.71 (0.64–0.79) ^3^	Wang et al., 2016 [166]
miR-125b, miR-126, miR-221 ^5^											N/A	
miR-572	Up	Plasma	Microarray, RT-qPCR	T2DM (50) HC (50)	Han Chinese	46.22 ± 6.90 45.52 ± 6.22	27/23 22/28	25.41 ± 0.32 25.36 ± 0.38	Newly diagnosed	8.69 ± 0.36 5.41 ± 0.29	0.843 (0.766–0.920) 87.8; 71.4	Yan et al., 2016 [179]
miR-320b	Down										0.946 (0.906–0.985) 92.0; 85.7	
miR-1249											0.784 (0.685–0.883) 86.0; 77.55	
miR-15a	Down	Whole blood	RT-qPCR	T2DM (24) HC (24)	Bahrainis	52 ± 6.0 49 ± 9.1	10/14 13/11	25.3 ± 1.8 24.2 ± 1.0	Diagnosed	7.5 ± 0.8 4.8 ± 0.6	0.864 (0.751–0.977)	Al Kafaji et al., 2015 [222]
miR-34c-5p, miR-576-3p	Up	PBMC	Microarray, RT-qPCR	T2DM (64) HC (44)	Ecuadorian	61 (37–85) 53 (32–87)	24/40 13/31	29.5 (22–49) 28.7 (23–42)	Diagnosed	7.0 (3.2–12.5) 5.6 (3.9–6.9)	N/A	Baldeón et al., 2015 [181]
miR-185	Down	Plasma	qPCR	T2DM (34) HC (30)	Mongolian (Chinese)	N/A	N/A	N/A	Diagnosed	N/A	N/A	Bao et al., 2015, [215]
miR-142, miR-143, miR-155, miR-223	Down	Platelet	RT-qPCR	T2DM (22) HC (22)	German	45.7 ± 3.1 41.6 ± 7.5	10/12 10/12	N/A	Diagnosed	9.01 ± 0.37 4.98 ± 0.58	N/A	Elgheznawy et al., 2015 [148]
miR-101, miR-375, miR-802	Up	Serum	sRNA-Seq (mice), RT-qPCR	T2DM (155) HC (49)	Japanese	62.3 ± 13.2 46.0 ± 9.67	96/59 25/24	25.9 ± 4.97 23.6 ± 4.05	Diagnosed	7.31 ± 1.08 6.03 ± 0.39	N/A	Higuchi et al., 2015 [167]
miR-10b, miR-130a, miR-143	Down	Whole blood	Microarray, RT-qPCR	T2DM (12) HC (24)	Xinjiang Uygurian	56 ± 10 49 ± 13	N/A	30.9 ± 5.8 26.3 ± 3.6	Diagnosed	N/A	N/A	Jiao et al., 2015 [182]
miR-146a ^6^	Down	PBMC	qPCR	T2DM (35) HC (35)	Indian	47.3 ± 7 44.7 ± 6	19/16 17/18	24.6 ± 2 23.9 ± 2	Diagnosed	7.8 ± 1.5 5.5 ± 0.4	N/A	Lenin et al., 2015 [204]
miR-103b	Down	Platelet	RT-qPCR	T2DM (43) HC (46)	Han Chinese	59 ± 9.3 51.4 ± 9.4	19/24 17/29	23.3 ± 5.4 21.9 ± 2.9	Newly diagnosed	7.0 ± 1.3 5.1 ± 0.5	N/A	Luo et al., 2015 [210]
miR-155	Down	PBMC	RT-qPCR	T2DM (20) HC (20)	Iranian	46.5 ± 5.8 47.5 ± 4.4	10/10 10/10	28.7 ± 4.9 26.2 ± 4.0	Diagnosed	7.02 ± 0.5 5.7 ± 0.7	N/A	Mazloom et al., 2015 [209]
miR-21-5p, miR-126-3p	Down	Plasma	RT-qPCR	T2DM (76) HC (107)	Italian	65.56 ± 6.96 64.25 ± 7.56	36/40 49/58	28.47 ± 4.34 26.67 ± 5.4	Diagnosed	7.34 ± 1.28 5.96 ± 0.41	N/A	Olivieri et al., 2015 [203]
miR-130b-3p, miR-374a-5p	Up	Serum	miRNA PCR assay, RT-qPCR	T2DM (49) HC (49)	Asian Indian	44.4 ± 8.1 44.3 ± 6.9	25/24 26/23	25.7 ± 3.5 24.5 ± 2.6	Newly diagnosed	7.8 ± 1.6 5.6 ± 0.4	N/A	Prabu et al., 2015 [168]
miR-126	Down	Plasma	qPCR	T2DM (20) HC (20)	Han Chinese	61.20 ± 10.62 57.25 ± 9.64	13/7 9/11	24.53 ± 2.87 23.90 ± 2.34	Newly diagnosed	N/A	0.806 77.78; 66.67	Zhang et al., 2015 [197]
miR-146a	Down	Serum	RT-qPCR	T2DM (56) HC (40)	Ecuadorian	62 (38–85) 54 (32–87)	22/34 12/28	29.2 (22–39) 29.3 (23–42)	Diagnosed	7.1 (4.8–12.5) 5.7 (3.9–6.7)	N/A	Baldeón et al., 2014 [202]
miR-126	Down	Serum	RT-qPCR	T2DM (160) HC (138)	Chinese	50.2 ± 6.7 46.7 ± 7.2	78/82 67/71	23.32 ± 0.31 22.87 ± 0.32	Newly diagnosed	9.16 ± 1.64 4.69 ± 0.57	0.792 (0.707–0.877)	Liu et al., 2014 [196]
miR-140-5p, miR-142-3p ^3^, miR-222	Up	Plasma	Microarray, RT-qPCR	T2DM (48) HC (45)	Spanish	54 ± 10 ^7a^ 57.7 ± 8 ^7b^ 48.1 ± 10.1 ^8a^ 50.6 ± 14.4 ^8b^	Only male	26.4 ± 2.4 ^7a^ 33.4 ± 3.3 ^7b^ 25.2 ± 1.8 ^8a^ 32.2 ± 2.4 ^8b^	Diagnosed	7.67 ± 1.46 ^7a^ 7.06 ± 2.14 ^7b^ 4.73 ± 0.35 ^8a^ 4.81 ± 0.33 ^8b^	0.975 ^3^	Ortega et al., 2014 [169]
miR-125b, miR-126 ^3^, miR-130b, miR-192, miR-195 ^3^, miR-423-5p ^3^, miR-532-5p	Down											
miR-326	Up	Plasma (exosomes)	Microarray, RT-qPCR	T2DM (18) HC (12)	Italian	57.2 ± 9.6 49.5 ± 12.4	12/6 6/6	31.6 ± 5.1 32.9 ± 5.4	Newly diagnosed	9.6 ± 1.5 5.7 ± 0.5	0.912 (0.799–1.000) ^3^	Santovito et al., 2014 [170]
let-7a, let-7f	Down											
miR-375	Up	Plasma	RT-qPCR	T2DM (100) HC (100)	Chinese Kazak	51.33 ± 11.75 48.55 ± 12.41	54/46 44/56	26.30 ± 4.08 24.44 ± 4.63	Diagnosed	N/A	N/A	Sun et al., 2014 [184]
miR-199a	Up	Plasma	RT-qPCR	T2DM (64) HC (64)	Han Chinese	46–62	N/A	N/A	Newly diagnosed	N/A	N/A	Yan et al., 2014 [186]
miR-23a	Down	Serum	Solexa sequencing, RT-qPCR	T2DM (24) HC (20)	Han Chinese	51.13 ± 9.21 46.65 ± 16.18	16/8 8/12	25.27 ± 2.90 25.55 ± 5.27	Newly diagnosed	9.49 ± 2.45 5.98 ± 0.80	0.835 (0.717–0.954) 79.2; 75.0	Yang et al., 2014 [171]
miR-486											0.698 (0.540–0.856) 79.2; 60.0	
let-7i											0.771 (0.629–0.913) 75.0; 70.0	
miR-96, miR-146a, miR-186, miR-191, miR-192											N/A	
miR-146a, miR-155	Down	PBMC	RT-qPCR	T2DM (20) HC (20)	Méxican	40–60 18–28	11/9 11/9	31.9 ± 7.4 23.1 ± 2.5	0–20	7.9 ± 1.7 4.8 ± 0.7	N/A	Corral-Fernández et al., 2013 [213]
miR-146a	Up	Plasma	RT-qPCR	T2DM (90) HC (90)	Han Chinese	48.5 (42.0–56.0) 48.00 (41.8–55.0)	47/43 47/43	24.58 ± 3.66 23.38 ± 2.95	Newly diagnosed	N/A	0.725 (0.651–0.799)	Rong et al., 2013 [195]
miR-126	Down	Plasma	qPCR	T2DM (30) HC (30)	Han Chinese	63 ± 8.6 61 ± 9	16/14 16/14	N/A	Newly diagnosed	N/A	N/A	Zhang et al., 2013 [201]
miR-27a, miR-150, miR-192, miR-320a	Up	Whole blood	Microarray, RT-qPCR	T2DM (29) HC (29)	Singaporean	44.2 ± 8.4 45.7 ± 11.3	N/A	26.5 ± 5.9 23.7 ± 3.2	Newly diagnosed	N/A	N/A	Karolina et al., 2012 [172]
miR-17, miR-92a, miR-130a, miR-195, miR-197, miR-509-5p, miR-652	Down											
miR-146a	Down	PBMC	qPCR	T2DM (20) HC (20)	Indian	43.7 ± 5.1 42.0 ± 4.7	N/A	26.4 ± 3.7 25.8 ± 4.0	Diagnosed	7.9 ± 1.8 5.5 ± 0.2	N/A	Balasubramanyam et al., 2011 [208]
miR-29a, miR-144, miR-150, miR-192, miR-320	Up	Whole blood	Microarray, RT-qPCR	T2DM (8) HC (7)	Singaporean	46.7 ± 3.4 46.3 ± 7.5	Only male	24.5 ± 1.1 22.4 ± 2.3	Diagnosed	N/A	N/A	Karolina et al., 2011 [180]
miR-30d, miR-146a, miR-182	Down											
miR-29a, miR-144, miR-150, miR-192, miR-320	Up			T2DM (13) HC (8)		41.0 ± 12.1 43.3 ± 5.7		28.0 ± 4.9 24.4 ± 3.1	Newly diagnosed			
miR-30d, miR-146a, miR-182	Down											
miR-9, miR-29a, miR-30d, miR-34a, miR-124a, miR-146a, miR-375	Up	Serum	RT-qPCR	T2DM (18) HC (19)	Han Chinese	47.33 ± 2.62 41.00 ± 2.62	9/9 12/7	26.26 ± 0.79 26.63 ± 0.80	Newly diagnosed	N/A	N/A	Kong et al., 2011 [183]
miR-28-3p	Up	Plasma	Microarray, RT-qPCR	T2DM (80) HC (80)	Italian (Bruneck cohort)	66.3 ± 8.9 66.3 ± 8.9	30/50 30/50	28.0 ± 4.4 25.0 ± 4.0	Diagnosed	6.5 ± 1.4 5.4 ± 0.3	N/A	Zampetaki et al., 2010 [174]
miR-15a, miR-20b, miR-21, miR-24, miR-29b, miR-126, miR-150, miR-191, miR-197, miR-223, miR-320, miR-486	Down											

^1^ gender distribution assessed for both studied and control groups; ^2^ miRNA assessed for 39 patients from control group; ^3^ values for miRNA panel; ^4^ miRNA assessed for 68 patients from both T2DM and HC groups; ^5^ miRNA assessed for 31 patients from both T2DM and HC groups; ^6^ miRNA assessed for 15 patients from both T2DM and HC groups; ^7a^ non-obese T2DM patients; ^7b^ obese T2DM patients; ^8a^ non-obese controls; ^8b^ obese controls. miRNA—microRNA; T2DM—type 2 diabetes mellitus; HC—healthy controls; PBMC—peripheral blood mononuclear cell; MPs—microparticles; qPCR—quantitative real-time polymerase chain reaction; RT-qPCR—reverse transcription quantitative real-time polymerase chain reaction; sRNA-Seq—small RNA sequencing; TLDA—TaqMan Low Density Array; BMI—body mass index; HbA1c—glycated hemoglobin A1c; AUC—area under the curve; 95% CI—95% confidence interval; SV—sensitivity; SP—specificity; NS—non-significant; N/A—not assessed; N/D—no data.

### 4.2. MicroRNAs as Potential Biomarkers for CAD

CAD is one of the most common cardiovascular diseases, caused by atherosclerotic plaque accumulation in the epicardial coronary arteries that lead to a different degree of stenosis or obstruction, which consequently restrict blood flow to the myocardium [243,244]. The disease can be stable and asymptomatic for a long time. Nevertheless, its progressive nature makes it serious due to the risk of an acute atherothrombotic event associated with plaque rapture or erosion that may occur even in clinically apparently silent periods [244,245]. Clinically, CAD can be classified into two subsets as acute coronary syndromes (ACS) and chronic coronary syndromes (CCS), previously known as stable CAD [244].

The diagnosis of stable CAD is often delayed because non-invasive diagnostic methods, including resting electrocardiogram and functional imaging, may be without any significant abnormalities [244]. Currently, in spite of the rising significance of cardiac computed tomography angiography, coronary angiography still remains the gold diagnostic standard to anatomically confirm CAD, but it is an invasive, expensive medical procedure associated with the risk of severe complications [244,246]. Therefore, extensive research on non-invasive, blood-based biomarkers is highly necessary for the early detection of CAD and the prevention of its further phases such as unstable angina, myocardial infarction, and sudden cardiac death [244].

Fichtlscherer et al. were the first to investigate the potential role of circulating miRNAs as biomarkers in patients with stable CAD [247]. The study has revealed that mainly endothelial-related miRNAs exerted significant differences in CAD individuals compared with controls [247]. Eight differentially expressed miRNAs, identified in microarray-based miRNA profiling, were validated in larger cohorts, and these data demonstrated that endothelial-enriched miRNAs miR-126, miR-17, and miR-92a, VSMC-enriched miR-145, inflammatory cell-enriched miR-155 were significantly reduced in both serum and plasma of patients with CAD, whereas cardiomyocyte-enriched miR-133 was elevated only in plasma [247]. These findings suggest that miRNA expression patterns may be distinctive between particular peripheral blood compartments, including serum, plasma, and PBMCs [247,248,249]. Additionally, this research indicates the need to perform pilot miRNA profiling to obtain a set of miRNAs exhibiting the most significant population-specific changes, which is why other ten studies have assessed the differences in hundreds of miRNAs, using mostly microarray profiling and NGS in a single case, in stable CAD as compared to non-CAD subjects [250,251,252,253,254,255,256,257,258,259].

Although many miRNAs show a satisfactory power to discriminate CAD patients from healthy ones as a single parameter [260,261,262,263,264,265,266], it is pertinent to note that the combination of several miRNAs can improve their diagnostic utility [252,253,257,259,267,268,269,270]. Interestingly, a 3-miRNA signature, consisting of miR-29a-3p, miR-574-3p, and miR-574-5p, has been proposed as a reliable marker for the diagnosis of CAD with AUC estimated at 0.916, which turned out to be higher than those calculated for each single miRNAs (0.830, 0.792, and 0.789, respectively) [267]. Similarly, a panel of four plasma-derived miRNAs, including let-7i-5p, miR-26a-5p, miR-32-3p, and miR-3149, has appeared quite promising in distinguishing between CAD and non-CAD patients [252]. The combination of these four miRNAs exhibited better diagnostic performance compared with any individual miRNA, with an AUC of 0.837 [252]. In the study conducted by Dong et al., a set of four lipometabolism-related miRNAs (miR-24, miR-33a, miR-103a, miR-122) isolated from PBMCs revealed the high discriminatory performance for CAD with 84.5% sensitivity and 81.9% specificity (AUC = 0.911) [269]. What is more, all of these parameters, when analyzed together, had better diagnostic accuracy with respect to sensitivity (69.6%, 72.0%, 64.6%, 68.3%) and specificity (65.1%, 67.1%, 60.4%, 65.1%) assessed for every single miRNA [269].

Considering that advanced age is a relevant risk factor for CAD development, the discovery of age-related miRNAs as stable CAD biomarkers seems to be promising due to the atypical symptomatology of the disease and the limitation of invasive diagnostics in elderly patients [271]. It has occurred that upregulated miR-765 had a significant association with the aging of the heart and may be a useful tool in the detection of CAD in the geriatric population (AUC = 0.959) [272]. In turn, the downregulation of serum miR-145-3p, miR-190a-5p, miR-196b-5p, miR-3163-3p, and upregulation of platelet-derived miR-340, miR-624, and endothelial-related miR-451b can help to discriminate patients with atypical, early-onset CAD diagnosed at a young age (at or before 55 years in men or 65 years in women) thus improving the prevention strategies [254,259,273]. Furthermore, Ali Sheikh et al. have revealed that alterations in miR-149, miR-424, and miR-765 expressions might be novel, sensitive (71.8%, 68.7%, 81.5%), and specific (95.3%, 92.3%, 93.7%) predictors for the diagnosis of middle-aged CAD patients [274]. In addition, miR-126 and miR-143 may serve as independent risk factors of CAD [275].

The growing role of miRNAs in the regulation of genes involved in the initiation and progression of coronary occlusion indicates that miRNAs may serve not only as diagnostic biomarkers but also to determine the severity and complexity of CAD. Currently, the extension of coronary atherosclerotic lesions is estimated according to the Gensini score (GS), and the Synergy between Percutaneous Coronary Intervention with Taxus and Cardiac Surgery (SYNTAX) score during invasive coronary angiography [268,276,277]. It has been proved that miR-17-5p (*r* = 0.489), miR-34a (*r* = 0.327), miR-133a (*r* = 0.303), miR-155 (*r* = 0.612), miR-208a (*r* = 0.853), miR-223 (*r* = 0.729), and miR-2909 (*r* = 0.943) expressions gradually increase with the severity of coronary occlusion reflected by GS, whereas downregulated expressions of miR-16 (*r* = −0.514), miR-126 (*r* = −0.351 or *r* = −0.416 according to different authors), miR-210 (*r* = −0.367), and miR-378 (*r* = −0.235 or *r* = −0.422 based on two independent studies), were negatively correlated with GS in patients with CAD (all *p* < 0.05) [249,264,268,277,278,279,280,281,282,283,284]. Moreover, miR-101a and miR-126-5p were significantly reduced in progressing stages of CAD in accordance with the SYNTAX score. Thus they may be considered biomarkers for evaluating the presence and severity of CAD [276,285]. It has also been reported that miR-145 negatively correlated with higher SYNTAX scores, indicating decreased plasma miR-145 expression with an increase in the severity of CAD [286]. Surprisingly, miR-33 showed notable differences only between the mild form of CAD (SYNTAX score ≤ 22) compared to controls, however, the authors have suggested that it may be the result of the low number of subjects analyzed in case of moderate and severe CAD [287]. That is why the possibility for the role of miR-33 at later stages of CAD cannot be ruled out [287]. Additionally, it has appeared that changes in the expressions of miR-23a, miR-27a, miR-126-5p, and miR-206 were associated with the number of vessels with angiographically documented atherosclerosis [285,288,289,290].

Interestingly, it has occurred that additional use of miR-223 may increase the diagnostic value of established cardiovascular risk factors, including LDL-C, HDL-C, and TG [264]. Similarly, Li et al. displayed a positive correlation between miR-34a and TC, LDL-C, and TG in patients suffering from CAD, highlighting that higher miR-34a expression and LDL-C levels with lower HDL-C levels were independently linked with increased CAD risk [282]. Anyway, a panel of these three parameters has been found to predict CAD risk with AUC greater than those calculated for miRNA alone (AUC = 0.912 vs. AUC = 0.899) [282]. It is worth noting that miR-2909 increases lipid peroxidation and ox-LDL uptake, thereby contributing significantly to the initiation and progression of the atherogenic process in individuals with CAD [277]. Among other miRNAs considered potential biomarkers for stable CAD, miR-20a, miR-30e, miR-92a, miR-101a, miR-122, miR-133a-5p, miR-144-3p, miR-222-5p, and miR-223 are involved in the regulation of cholesterol metabolism [276,291,292,293,294]. Apart from being closely associated with blood lipids, several miRNAs, including miR-16, miR-34a, miR-126, miR-342-5p, and miR-2909, also disclose a correlation with inflammatory- (C-reactive protein, TNF-α, interferon-γ (IFN-γ), IL-1β, IL-6, IL-8, IL-10, IL-17, VCAM-1, ICAM-1) and oxidative stress-related indexes (ROS) [277,279,281,282,295]. The studies showing the potential relevance of miRNAs as biomarkers for stable CAD are summarized in Table 2.

### 4.3. MicroRNAs as Potential Biomarkers for CAD Related to T2DM

Accumulating evidence has demonstrated that patients with T2DM are more prone to develop subsequent CAD than individuals without diabetes [296,297]. It is worth noting that T2DM shares a number of well-established risk factors with CAD, involving mainly dyslipidemia, hypertension, and obesity [297,298]. These cardiometabolic features, along with coexisting chronic low-grade inflammation, hypercoagulability, and increased oxidative stress under a hyperglycemic milieu, may contribute to the approximate doubling of CAD risk in patients with T2DM [298]. It has led to the ‘common soil’ hypothesis, postulating that both conditions have a common pathogenetic background [296]. Thus, revealing the underlying molecular mechanism of T2DM promoting the pathological progression of cardiovascular disease will not only help to alleviate the cardiovascular damage caused by diabetes-accelerated atherosclerosis but also to identify the candidate molecules for stratifying the risk of CAD in T2DM.

**Figure 3:**
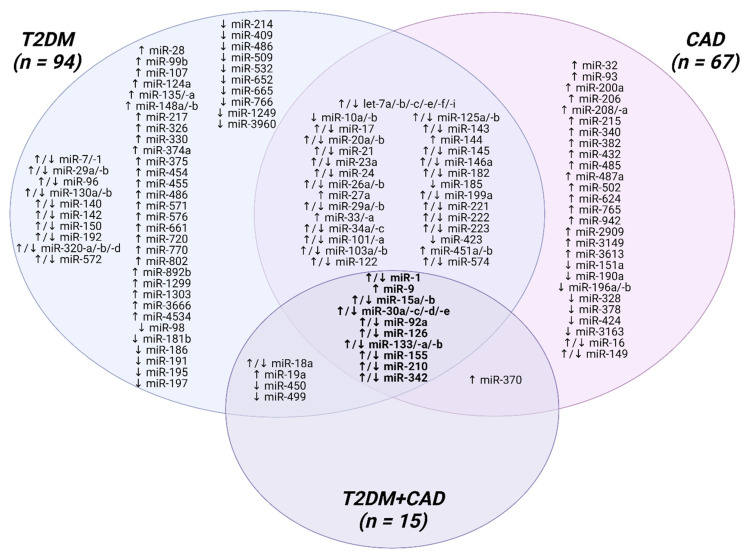
Venn diagram of differentially expressed microRNAs in patients with T2DM, CAD, and CAD related to T2DM. Overlapping fields indicate miRNAs that have been associated with two of these pathologies. In the center of the figure, ten miRNAs are highlighted with different expressions that have been detected in T2DM, CAD and T2DM with CAD. Created with BioRender.com.

To date, more than 120 studies (Table 1 and Table 2) have attempted to explore the potential of miRNAs as a diagnostic tool for T2DM or CAD in clinical practice. However, there are only a few reports on miRNA-based biomarkers for the early detection of CAD in asymptomatic patients with T2DM (Table 3). As shown in Figure 3, only ten of the 39 miRNAs overlapping in T2DM, and CAD were evaluated for their relevance in the detection of CAD in T2DM. Nonetheless, distinct sets of unique circulating miRNAs were also discovered for each disease (94 miRNAs for T2DM and 67 miRNAs for CAD).

One of the most thoroughly studied miRNAs in both T2DM and CAD is the anti-inflammatory miR-126, which is responsible for maintaining endothelial homeostasis and vascular integrity. The expression of miR-126 in whole blood and plasma has been observed to be remarkably decreased by almost 1.9–4.6-fold between T2DM patients with and without CAD and 7.7–13.1-fold between T2DM with CAD and healthy individuals [198,223]. The obtained intergroup differences allowed miR-126 to be proposed as a potential biomarker of CAD occurrence in T2DM patients with a discriminatory ability ranging from AUC 0.807 to 0.970, according to two different studies [198,223]. Moreover, miR-126 showed a negative correlation with the concentration of LDL-C (*r* = −0.575, *p* < 0.0001 or *r* = −0.46, *p* = 0.001), TC (*r* = −0.48, *p* = 0.001), fasting plasma glucose (*r* = −0.92, *p* < 0.001), HbA1c (*r* = −0.81, *p* < 0.001), whereas a positive one was observed in case of HDL-C (*r* = 0.41, *p* = 0.005), which directly proves association of this miRNA with known risk factors of CAD in T2DM [198,223]. It is worth emphasizing that patients with simultaneously reduced expression of miR-126 and HDL-C levels were more likely to develop diabetes complicated by CAD [223]. In addition, it has been reported that individuals with T2DM exhibiting lower levels of miR-126 were at a higher risk of comorbid CAD and previous major cardiovascular events compared to those subjects suffering from other diabetes-related complications [203,299].

According to recent scientific reports, other promising candidates, miR-92a, miR-342, and miR-450, seem to play a beneficial role in the prediction of CAD in patients with T2DM because, such as miR-126, they are strongly involved in inflammatory and oxidative stress pathways [233,300]. Seelem et al. have revealed significant upregulation of miR-342 expression and downregulation of miR-450 expression in the serum of individuals with T2DM, CAD, and T2DM-related CAD compared to controls [233]. Additionally, these miRNAs displayed a proatherogenic nature due to the observed valid correlations with dyslipidemic (TG, TC, LDL-C, HDL-C), anthropometric (BMI, waist-to-hip ratio; WHR) variables, and glucose homeostasis indices (HbA1c, fasting plasma glucose, diabetes duration) [233]. Interestingly, miR-342 and miR-450 were associated with the activity of NADPH oxidase 4 (NOX-4), an enzyme involved in promoting ROS production and CRP concentration, unrevealing the underlying molecular pathomechanisms during the development of CAD in patients with T2DM [233]. Both miR-342 and miR-450 demonstrated good or very good discriminatory power for CAD in T2DM (AUC = 0.781, 80.0% sensitivity, 72.0% specificity and AUC = 0.824, 72.0% sensitivity, 78.0% specificity, respectively) [233]. In turn, Wang et al. have explored miR-92a as a potential biomarker for CAD in diabetes and a contributor to CAD onset through the activation of NF-κB, a critical regulator of inflammation, and its downstream inflammatory pathways [300]. Progressively increasing expression of miR-92a in serum from healthy individuals via T2DM to T2DM with CAD showed positive correlations with NF-κB p65 expression and the level of proinflammatory cytokines, including ET-1, MCP-1, and ICAM-1, in patients with T2DM and CAD [300]. What is more, logistic regression analysis revealed a strong association of miR-92a expression with CAD in T2DM, showing an OR of 15.835 (95% CI, 6.307–39.754; *p* < 0.001), thus indicating miR-92a as an independent risk factor for T2DM-related CAD [300]. miR-92a may help predict CAD in T2DM with a calculated AUC of 0.866 (76.9% sensitivity and 88.4% specificity) [300].

It is pertinent to note that individuals with T2DM are characterized by abnormal fibrinolytic activity with elevated levels of PAI-1, known as an independent risk factor for hypercoagulable and thrombotic events in this group of patients [224]. Luo et al. have observed decreased expression of plasma miR-30c in T2DM patients with CAD as compared to those without this vascular complication [224]. Furthermore, miR-30c negatively correlated with circulating PAI-1 (*r* = −0.733, *p* < 0.0001) and the degree of coronary artery lesions evaluated by the GS criteria (*r* = −0.782, *p* < 0.0001) in the T2DM with CAD group [224]. Interestingly, patients with T2DM and CAD had more advanced coronary lesions, even than those diagnosed with CAD, supporting the hypothesis of T2DM-accelerated atherosclerosis [224]. In line with ROC analysis results, miR-30c exerted excellent capability to distinguish T2DM-CAD from healthy controls with an AUC of 0.972 (90.9% sensitivity and 85.2% specificity) [224]. Yet, the diagnostic value of miR-30c in the early detection of CAD among T2DM patients occurred to be poor (AUC of 0.474, 70.2% sensitivity and 52.0% specificity) [224].

Regarding miRNAs as possible diagnostic biomarkers for CAD in highly susceptible individuals with T2DM, it is relevant to consider especially those miRNAs that strictly correspond to their origin and the injury of a given tissue. So far, three cardiomyocyte-enriched miRNAs, miR-1, miR-133, and miR-499, revealed a good or very good discriminatory power to identify CAD in patients with T2DM [226,301]. Although several miRNAs having obtained good performance in detecting CAD in T2DM patients, it should be mentioned that miRNAs may target multiple genes and regulate different signaling pathways. Therefore, a single miRNA might be insufficient for diagnostic purposes, and the miRNA panels can better reflect the complex pathophysiology of T2DM-related CAD. It turned out that the combined signature composed of two upregulated miRNAs, miR-9 and miR-370, demonstrated higher sensitivity and specificity (84.0%, 84.0%) in the identification of CAD in T2DM compared to each miRNA alone (72.0% sensitivity, 82.0% specificity and 26.0% sensitivity, 82.0% specificity, respectively) [302]. Although the two-miRNA panel consisting of miR-1 and miR-133 achieved good discriminatory performance for CAD in T2DM (AUC = 0.752), the diagnostic value for miR-1 (AUC = 0.802) alone was even better than for the miRNA panel, probably due to the only marginally statistically significant AUC for miR-133 [301].

So far, only Zhang et al. have aimed to explore miRNA expression patterns in T2DM-CAD patients and identify novel miRNA molecules as disease biomarkers [303]. Based on miRNA profiling, five downregulated EV-carried miRNAs (miR-15a-3p, miR-18a-5p, miR-133a-3p, miR-155-5p, miR-210-3p) and upregulated miR-19a-3p have been established as promising biomarkers for CAD related to T2DM with satisfactory or even excellent values of AUC [303].

As mentioned previously, a distinct miRNA profile may be observed depending on the body fluids used. It has been demonstrated that miR-210 (downregulated in EVs or upregulated in plasma according to different studies) showed better predictive value when assessed in plasma (AUC = 0.786 vs. AUC = 0.980, respectively), therefore further research is needed to determine the exact blood compartment for future clinical evaluation [223,303].

**Table 3:** Circulating microRNAs as potential biomarkers for stable coronary artery disease related to type 2 diabetes mellitus.

miRNA	Expression Change	Sample Type	Assay Method	Number of Samples	Ethnicity	Age [Years]	Gender (Male/Female, *n*)	BMI [kg/m^2^]	Duration of T2DM [Years]	HbA1c [%]	Value of Biomarker AUC (95% CI); SV [%]; SP [%]	Author, Year (Reference)
miR-499	Down	Serum	RT-qPCR	T2DM+CAD (60) T2DM (60) HC (60)	Egyptian	Age-matched	Sex-matched	BMI-matched	N/A	N/D	0.720 73.0; 70.0	Oraby et al., 2022 [226]
miR-19a-3p	Up	Plasma (EVs)	sRNA-Seq, RT-qPCR	T2DM+CAD (32) HC (20)	Chinese	Age-matched	Sex-matched	N/A	N/A	N/D	0.698 (0.530–0.866) ^2^ 53.9; 85.7	Zhang et al., 2022 [303]
miR-15a-3p	Down										0.874 (0.765–0.982) ^2^ 88.5; 71.4	
miR-18a-5p											0.871 (0.760–0.982) ^2^ 80.8; 78.6	
miR-133a-3p											0.745 (0.567–0.922) ^2^ 88.5; 57.1	
miR-155-5p											0.901 (0.800–1.000) ^2^ 92.3; 78.6	
miR-210-3p											0.786 (0.647–0.925) ^2^ 61.5; 92.9	
miR-1, miR-133	Up	Whole blood	RT-qPCR	T2DM+CAD (30) T2DM (30) HC (30)	Bahrainis	60 ± 12 58 ± 11.5 56 ± 5.1	15/15 12/18 14/16	25.35 ± 4.4 25.7 ± 5.2 24.2 ± 4.6	15 ± 4.4 14 ± 9.3 –	8.68 ± 2.6 7.09 ± 1.06 5.03 ± 0.7	0.752 (0.626–0.879) ^1,3^	Al-Muhtaresh et al., 2019 [301]
miR-1											0.912 (0.828–0.995) ^2^	
miR-133											0.920 (0.842–0.998) ^2^	
miR-30c	Down	Plasma	qPCR	T2DM+CAD (27) T2DM (47) CAD (34) HC (32)	Han Chinese	64.5 ± 6.5 60.5 ± 11.1 60.9 ± 5.3 58.6 ± 8.1	17/10 23/24 18/16 17/15	25.02 ± 3.12 24.76 ± 3.29 24.57 ± 3.01 24.49 ± 2.30	Newly diagnosed	9.13 ± 1.01 9.15 ± 1.02 6.28 ± 0.69 5.36 ± 0.35	0.474 (0.355–0.593) ^1^ 70.2; 52.0 0.972 (0.940–1.000) ^2^ 90.9; 85.2	Luo et al., 2019 [224]
miR-342	Up	Serum	RT-qPCR	T2DM+CAD (50) T2DM (50) CAD (50) HC (50)	Egyptian	62.30 ± 0.61 62.06 ± 1.26 62.32 ± 0.56 62.22 ± 0.69	Only female	28.87 ± 0.33 27.58 ± 0.28 27.88 ± 0.23 23.82 ± 0.14	12.06 ± 0.30 12.06 ± 0.30 – –	11.92 ± 0.18 10.75 ± 0.17 9.73 ± 0.17 4.10 ± 0.68	0.781 ^1^ 80.0; 72.0	Seleem et al., 2019 [233]
miR-450	Down										0.824 ^1^ 72.0; 78.0	
miR-92a	Up	Serum	RT-qPCR	T2DM+CAD (117) T2DM (69) HC (68)	Chinese	64.73 ± 8.22 64.29 ± 3.77 62.98 ± 7.42	79/38 48/21 45/23	26.44 ± 3.31 25.61 ± 5.76 24.08 ± 2.42	N/A	8.22 ± 2.64 6.80 ± 2.41 5.29 ± 0.33	0.866 ^1^ 76.9; 88.4 0.958 ^2^ 78.6; 98.5	Wang et al., 2019 [300]
miR-210	Up	Plasma	RT-qPCR	T2DM+CAD (46) T2DM (54) HC (20)	Egyptian	57.0 ± 6.2 56.5 ± 7.7 58.1 ± 1.1	23/23 29/25 11/9	29.7 ± 3.5 30.7 ± 5.3 23.2 ± 0.2	11.2 ± 5.2 10.8 ± 7.8 –	9.4 ± 1.0 8.3 ± 1.1 4.8 ± 0.4	0.980 ^1^ 93.5; 100.0 0.980 ^2^ 97.8; 100.0	Amr et al., 2018 [223]
miR-126	Down										0.970 ^1^ 91.3; 100.0 0.980 ^2^ 97.8; 95.0	
miR-126	Down	Whole blood	RT-qPCR	T2DM+CAD (45) T2DM (45) HC (45)	Bahrainis	64 ± 11.7 61 ± 12 53 ± 8.6	24/21 23/22 21/24	26.1 ± 4.3 25.4 ± 4.8 24 ± 4.5	18 ± 9.3 16 ± 6 –	9.6 ± 3.2 7.4 ± 8.3 3.64 ± 1.1	0.807 (0.714–0.900) ^1^ 0.948 (0.894–1.000) ^2^	Al-Kafaji et al., 2017 [198]
miR-9 ^4^, miR-370	Up	Serum	RT-qPCR	T2DM+CAD (50) T2DM (50) CAD (50) HC (50)	Egyptian	62.30 ± 0.45 62.06 ± 1.26 62.32 ± 0.56 62.22 ± 0.69	35/15 32/18 38/12 36/14	28.87 ± 0.32 27.58 ± 0.27 27.88 ± 0.23 23.82 ± 0.13	12.06 ± 0.30 12.22 ± 0.30 – –	11.0–12.5 ^5^	N/A 84.0; 84.0 ^3^	Motawae et al., 2015 [302]

^1^ value of miRNA to distinguish T2DM+CAD patients from T2DM ones; ^2^ value of miRNA to distinguish T2DM+CAD patients from HC; ^3^ values for miRNA panel; ^4^ significant differences were observed between T2DM+CAD patients and those from both CAD and HC groups; ^5^ HbA1c assessed for all T2DM patients. miRNA—microRNA; T2DM—type 2 diabetes mellitus; CAD—coronary artery disease; T2DM+CAD—type 2 diabetes mellitus with coronary artery disease; HC—healthy controls; EVs—extracellular vesicles; qPCR—quantitative real-time polymerase chain reaction; RT-qPCR—reverse transcription quantitative real-time polymerase chain reaction; sRNA-Seq—small RNA sequencing; BMI—body mass index; AUC—area under the curve; 95% CI—95% confidence interval; SV—sensitivity; SP—specificity; N/A—not assessed; N/D—no data.

## 5. MicroRNAs as Potential Therapeutic Targets in T2DM and CAD

Given the emerging role of miRNAs in the regulation of several steps in the disease pathway, they may not only pave the way for novel diagnostic strategies in T2DM and CAD but also seem to be attractive therapeutic targets. The ability to modulate miRNA expression by repressing pathological miRNAs or overexpressing protective miRNAs has led to the development of therapies based on miRNA inhibitors, such as locked nucleic acid (LNA) antimiRs, antagomiRs, and miRNA sponges, or miRNA mimics, respectively [304]. Currently, miRNA inhibitors are the most common in vivo approach when designing miRNA-based therapies [304].

One of the pioneering studies on the potential application of antimiRs was the study conducted by Trajkovski et al., who administered a recombinant adenovirus expressing 2′O-methylmodified antimiR-103 and -107 (15 mg/kg for two consecutive days) to *ob/ob* mice [305]. It has been observed that this therapy leads to a decrease in plasma glucose and insulin levels and improves glucose tolerance and sensitivity, especially in the liver and adipose tissue [305]. Of note, a GalNAc-conjugated oligonucleotide targeting miR-103/-107 (RG-125, AZD4076) has entered phase I/IIa randomized, single-blind, placebo-controlled clinical trial (NCT02826525) investigating the safety and tolerability of AZD4076 and assessing its effect on insulin sensitivity and liver fat content in patients with T2DM and non-alcoholic fatty liver disease, although there are still no results in the available literature. Recent studies have demonstrated that intravenous, subcutaneous, or intraperitoneal injections with antimiR-34a, antagomiR-132, LNA-29, and LNA-181a antimiRs reduce blood glucose levels, decrease inflammation, improve insulin secretion and its hepatic sensitivity in a mouse model of obesity [306,307,308,309]. Interestingly, subcutaneous administration of antagomir-22 (APT-110) at a dose of 15 mg/kg on days 0, 2, 4 of week 1 of treatment and then once a week for a total of 8 or 12 weeks in mice with diet-induced obesity provides a sustained increase in energy expenditure, reduction in body mass of 30% and liver steatosis, decreases blood glucose, insulin, cholesterol, leptin levels, and alleviates insulin resistance [310,311]. Based on in vivo experimental studies (*db/db* mice, rats with streptozocin-induced T2DM), it turned out that long-term injections with LNA-21 or antagomiR-21 may reduce body weight, pericardial fat, adipocyte size, improve glucose homeostasis (decrease HOMA-IR, HbA1c, blood glucose concentration, elevate plasma adiponectin level) and lipid metabolism parameters [312,313]. Moreover, this potential miRNA-based drug has revealed no apparent liver and kidney toxicity nor negative effects on cardiac function [312]. Among the miRNA mimics tested so far in the T2DM animal model, miR-125a, miR-145, and miR-383 agomiRs have been found to be able to improve glucose tolerance and lipid homeostasis indices as well as decrease inflammation in T2DM [103,314,315,316].

In vivo studies have also indicated that the regulation of miRNA expression may influence the development and progression of atherosclerosis and, thus, CAD. It has been observed that subcutaneous or intravenous administration of antagomiR-155 to mice with atherosclerosis leads to a reduction in macrophages in atherosclerotic plaque, suppresses its development, and decreases the level of serum inflammatory markers without significant effect on blood lipids and body weight [317,318]. Moreover, Wei et al. have revealed diminished macrophage and VSMC content in atherosclerotic plaque after injection of LNA-342 antimiR (25 mg/kg), which also inhibited miR-155 expression [319]. Similar effects have been obtained after intravenous administration of antagomiR-133b, resulting in a wider vascular lumen, more stable plaque size, thicker fibrous cap, smaller lipid core, and reduced macrophage immune response [320]. Another tested miRNA-based therapy with a potential vasoprotective effect in both diabetic and atherosclerotic mice is antagomiR-92a, which reduces oxidative stress and the content of macrophages, T lymphocytes in atherosclerotic plaque, but without a positive impact on lipid parameters [76,321,322]. Preclinical studies on the usefulness of antimiR-33 seemed promising, as short-term subcutaneous injections of antimiR-33 at a dose of 5–10 mg/kg led to a reduction in local and systemic inflammation as well as atherosclerotic plaque size with a simultaneous improvement in plaque stability or plasma HDL-C level [111,323,324,325,326]. Nevertheless, long-term antimiR-33 therapy might cause deleterious effects, including moderate steatosis and hypertriglyceridemia [327]. Furthermore, administration of antimiR-122, LNA-148a, or miR-30c mimic has been found to reduce TC, LDL-C, and TG levels, increase HDL-C level, diminish plaque size and hepatic steatosis, and prolong time to occlusion in in vivo mouse experimental models [151,328,329,330]. There are also several in vivo studies assessing the potential anti-inflammatory and atheroprotective ability of therapy based on antagomiR-17-5p, agomiR-188-3p, agomiR-200a, or agomiR-532-3p [331,332,333,334]. Interestingly, the administration of antagomiR-449a has revealed protective effects against atherosclerosis in diabetic mice [335].

Despite many studies that have been conducted in vivo on an animal experimental model of T2DM and CAD, there are still no ongoing registered clinical trials at clinicaltrials.gov which aimed to test miRNA-based therapeutics in humans. The clinical application of these promising next-generation drugs requires solving several critical issues, including the potential of miRNAs to interact with multiple pathways/targets, the risks of off-target toxicity, and the need for efficient and safe vectors or delivery systems to transport miRNA-regulating agents into target cells.

## 6. Conclusions and Future Directions

Over the past decade, an increasing number of reports have highlighted that aberrant expression of miRNAs may impair diverse signaling pathways underlying the pathophysiology of cardiometabolic diseases such as T2DM and CAD. Findings from preclinical evidence collected in this review strongly indicate both atheroprotective and atherogenic effects of different miRNAs targeting specific steps in the initiation and progression of hyperglycemia-induced atherosclerosis as a key player linking T2DM with CAD. Although a list of circulating miRNAs involved in T2DM and CAD development is still expanding, the existing data concerning their expression patterns are conflicting. Hence, to better evaluate the potential usefulness of miRNAs as diagnostic and prognostic tools in CAD related to T2DM, the group of candidate miRNA-based biomarkers in T2DM and CAD alone has also been provided. Among the 122 differentially expressed miRNAs that were analyzed in this paper, 14 top miRNAs appear to be the most consistently dysregulated miRNAs in T2DM (miR-135/-a, miR-148a/-b, miR-191, miR-195, miR-197, miR-375, miR-486, miR-766, miR-770) and CAD (miR-32, miR-206, miR-208/-a, miR-378, miR-765) posing them as promising biomarker candidates. miR-1, miR-9, miR-15a, miR-30, miR-92a, miR-126, miR-133a, miR-155, miR-210, and miR-342 were found to have the potential to arrive at miRNA-based risk stratification and early, non-invasive diagnosis of CAD among T2DM patients. Of note, these miRNAs are involved in the core processes associated with T2DM-induced atherosclerosis, mainly endothelial dysfunction, inflammation, VSMC proliferation/migration, and platelet hyperactivity. It may be a helpful suggestion for further studies in the search for novel miRNA-based biomarkers and therapeutic strategies in T2DM patients with CAD.

Nevertheless, the translation of these molecules into clinical practice faces many challenges. To avoid the discrepancies between miRNA expression profiles with generally poor reproducibility observed in available studies, establishing standardized miRNA detection technologies, efficient normalization, and validation methods are required. Based on the fact that miRNAs exhibit unique and stable patterns in various body fluids, determining the appropriate biological material (whole blood, plasma, serum, PBMCs, or saliva) for miRNA measurement, is necessary to incorporate miRNAs into clinical management. The ‘one gene, one protein, one disease’ paradigm may not fully reflect the complex pathophysiology of CAD related to T2DM. Thus, the use of a panel of two or more selected circulating miRNAs can be more effective and guarantee higher specificity and sensitivity in the detection of CAD among T2DM individuals. Therefore, the success of the implementation of miRNAs in a personalized approach to the diagnosis of T2DM-related CAD will mostly depend on further simultaneous studies in larger and well-defined populations to evaluate and strengthen their significance as promising biomarkers.

## Figures and Tables

**Table 2:** Circulating microRNAs as potential biomarkers for stable coronary artery disease.

miRNA	Expression Change	Sample Type	Assay Method	Number of Samples	Ethnicity	Age [Years]	Gender (Male/Female, *n*)	BMI [kg/m^2^]	Value of Biomarker AUC (95% CI); SV [%]; SP [%]	Author, Year (Reference)
miR-9-5p	Up	Serum	RT-qPCR	CAD (40) HC (20)	Iranian	42.36 ± 5.2 42.9 ± 4.6	19/21 4/16	N/A	0.693 (0.530–0.857) 74.07; 53.33	Gholipour et al., 2022 [260]
miR-182-5p									0.752 (0.608–0.897) 74.19; 66.67	
miR-27a	Up	Plasma	qPCR	CAD (30) HC (30)	Iranian	57.6 ± 20.32 55.30 ± 8.40	Only male	24.8–30.0 24.66–27.0	0.67 (0.54–0.81) 86.7; 46.7	Hosseinpor et al., 2022 [288]
miR-146a	Down								N/A	
miR-34a	Up	Plasma	RT-qPCR	CAD (203) HC (100)	Chinese	61.5 ± 9.4 62.0 ± 6.7	158/45 75/25	24.2 ± 3.0 23.8 ± 3.1	0.899 (0.865–0.934) 76.4; 90.0	Li et al., 2022 [282]
miR-122	Up	Serum	RT-qPCR	CAD (100) HC (100)	Indian	52.15 ± 1.13 50.90 ± 2.08	75/25 74/26	>26 (44.0%) >26 (33.0%)	0.806 64.0; 84.0	Ali et al., 2021 [261]
miR-126	Down								0.806 61.54; 80.0	
miR-200a-3p, miR-382-3p, miR-432-5p, miR-3613-3p	Up	Plasma (exosomes)	NGS	CAD (52) HC (52)	Han Chinese	65.04 ± 10.68 60.65 ± 11.26	43/9 34/18	26.87 ± 3.80 26.42 ± 3.57	N/A	Chang et al., 2021 [250]
miR-125a-5p, miR-151a-3p, miR-185-5p, miR-328-3p	Down									
miR-122	Down	Serum	RT-qPCR	CAD (78) HC (60)	Indian	52.07 ± 9.94 50.13 ± 8.12	N/A	25.9 ± 4.8 25.2 ± 4.7	N/A	Mishra et al., 2021 [291]
miR-101a	Down	Serum	RT-qPCR	CAD (200) HC (100)	Chinese	62 (31–87) ^1a^ 59 (37–80) ^1c^ 60 (40–86)	74/26 ^1a^ 78/22 ^1c^ 73/27	N/A	N/A	Yu et al., 2021 [276]
miR-23a, miR-27a	Up	PBMC	qPCR	CAD (82) HC (80)	Iranian	60.24 ± 0.91 57.40 ± 0.94	35/47 41/39	27.60 ± 0.48 26.23 ± 0.50	N/A	Babaee et al., 2020 [289]
miR-21	Up	Plasma	RT-qPCR	CAD (24) HC (54)	Indian	N/A	N/A	N/A	0.780 (0.670–0.890)	Kumar et al., 2020 [262]
miR-133b	Down			CAD (28) HC (54)					0.746 (0.620–0.870)	
miR-21	Up	PBMC	RT-qPCR	CAD (56) HC (29)	Turkish	58.96 ± 8.95 56.93 ± 6.35	41/15 9/20	N/A	N/A	Sanlialp et al., 2020 [248]
miR-155, miR-221	Down									
miR-10a-5p	Down	Serum	sRNA-Seq, RT-qPCR	CAD (39) HC (39)	Chinese	63.1 ± 7.4 59.3 ± 6.8	21/18 21/18	24.2 ± 2.3 23.2 ± 2.2	0.817 (0.715–0.918)	Wang et al., 2020 [251]
miR-423-3p									0.656 (0.532–0.779)	
miR-423-3p				CAD (30) HC (21)		63.9 ± 7.95 57.24 ± 5.35	15/15 9/12	24.57 ± 2.37 25.01 ± 2.02	0.808 (0.684–0.932)	
miR-16	Down	Plasma, PBMC	RT-qPCR	CAD (40) HC (40)	Chinese	63.33 ± 5.63 61.20 ± 5.82	Only male	26.69 ± 2.85 25.85 ± 3.12	N/A	Wang et al., 2020 [281]
miR-29a-3p, miR-574-3p, miR-574-5p	Up	Plasma	qPCR	CAD (88) HC (67)	Chinese	61.66 ± 1.32 63.72 ± 0.99	55/33 40/27	26.18 ± 0.50 24.89 ± 0.68	0.916 (0.856–0.957) ^2^	Zhang et al., 2020 [267]
let-7i-5p, miR-26a-5p, miR-32-3p, miR-3149	Up	Plasma	Microarray, RT-qPCR	CAD (40) HC (69)	Chinese	56.2 ± 7.6 55.0 ± 6.5	30/10 43/26	N/A	0.837 (0.763–0.911) ^2^	Zhang et al., 2020 [252]
miR-32-5p	Up	Serum (exosomes)	qPCR	CAD (20) HC (20)	Chinese	64 (52–68) 57 (52–62)	14/6 12/8	24.7 ± 2.9 24.0 ± 3.0	0.691 (0.525–0.858) 85.0; 55.0	Zhang et al., 2020 [263]
miR-149-5p									0.702 (0.536–0.869) 70.0; 75.0	
miR-942-5p									0.693 (0.527–0.858) 80.0; 60.0	
miR-133a-5p, miR-144-3p, miR-222-5p	Up	Plasma	RT-qPCR	CAD (46) HC (43)	Turkish	60.02 ± 10.01 55.26 ± 13.85	34/12 28/15	27.87 (25.04–30.43) 27.10 (24.38–29.42)	N/A	Gorur et al., 2019 [292]
miR-378	Down	Plasma	RT-qPCR	CAD (215) HC (52)	Chinese	61 ± 10 61 ± 12	153/62 30/22	23 ± 10 22 ± 10	0.789 (0.728–0.851)	Li et al., 2019 [283]
miR-451b	Up	Serum	RT-qPCR	CAD (30) HC (30)	Chinese	46–59 45–58	15/15 15/15	N/A	N/A	Lin et al., 2019 [273]
miR-30c	Down	Plasma	qPCR	CAD (34) HC (32)	Han Chinese	60.9 ± 5.3 58.6 ± 8.1	18/16 17/15	24.57 ± 3.01 24.49 ± 2.30	0.895 (0.811–0.978)	Luo et al., 2019 [224]
miR-33	Up	Plasma	RT-qPCR	CAD (30) HC (30)	Indian	54.7 ± 8.7 56.17 ± 9.18	15/15 15/15	26.67 ± 3.7 27.47 ± 4.35	N/A	Reddy et al., 2019 [287]
miR-30e, miR-92a	Up	Plasma (exosomes)	RT-qPCR	CAD (42) HC (42)	Chinese	63 63	22/20 22/20	N/A	N/A	Wang et al., 2019 [293]
miR-206	Up	Plasma	qPCR	CAD (100) HC (30)	Iranian	57 ± 9 55 ± 8	87/13 16/14	27.78 ± 3.45 27.45 ± 2.09	N/A	Zehtabian et al., 2019 [290]
miR-342-5p	Up	PBMC	qPCR	CAD (82) HC (80)	Iranian	60.10 ± 0.89 57.86 ± 0.97	35/47 41/39	27.56 ± 0.47 26.14 ± 0.49	0.702 (0.620–0.783)	Ahmadi et al., 2018 [295]
miR-20a, miR-92a, miR-223	Up	Plasma	RT-qPCR	CAD (19) HC (6)	Australian	65.2 ± 10.7 59.0 ± 5.1	19/0 5/1	N/A	N/A	Choteau et al., 2018 [294]
miR-223	Up	Plasma	RT-qPCR	CAD (300) HC (100)	Chinese	56.2	N/A	N/A	0.933 (0.905–0.961)86.0; 91.3	Guo et al., 2018 [264]
miR-155	Up	Serum	RT-qPCR	CAD (300) HC (100)	Chinese	N/A	N/A	N/A	N/A	Qiu et al., 2018 [249]
miR-126	Down	PBMC	qPCR	CAD (119) HC (96)	Chinese	59 ± 11 57 ± 10	36/83 27/69	24.6 ± 3.9 23.8 ± 3.4	0.801 (0.740–0.861) 70.6; 85.4	Wu at al., 2018 [279]
miR-221-3p	Down	Serum	qPCR	CAD (89) HC (93)	Turkish	58.97 ± 13.79 57.07 ± 9.80	N/A	NS	0.623 (0.539–0.702) 76.27; 49.43	Yilmaz et al., 2018 [265]
miR-222-3p									0.654 (0.571–0.731) 69.49; 54.02	
miR-17-5p, miR-92a, miR-126, miR-210, miR-378	Down	Plasma	RT-qPCR	CAD (102) HC (92)	Chinese	60.2 ± 11.4 57.9 ± 14.8	21/81 26/66	24.2 ± 3.7 23.6 ± 3.5	0.756 (0.687–0.725) ^2^ 84.3; 60.9	Zhang et al., 2018 [268]
miR-24, miR-33a, miR-103a, miR-122	Up	PBMC	RT-qPCR	CAD (161) HC (149)	Chinese	61.35 ± 7.10 61.08 ± 7.51	86/75 72/77	25.77 ± 3.06 24.81 ± 3.29	0.911 (0.880–0.942) ^2^ 84.5; 81.9	Dong et al., 2017 [269]
let-7c, miR-145, miR-155	Down	Plasma	OpenArray RT-qPCR, RT-qPCR	CAD (69) HC (32)	French (South-western)	58.4 ± 9.0 57.3 ± 11.6	Only male	27.3 ± 4.4 26.6 ± 3.1	0.708 (0.600–0.811) ^2^ 75.76; 63.33	Faccini et al., 2017 [253]
miR-126, miR-143, miR-145 ^3^	Up	PBMC	RT-qPCR	CAD (450) HC (450)	Chinese	< 60 (26.8%/25.6%) 60–80 (68.0%/67.8%) ≥ 80 (5.2%/6.7%)	234/216 215/235	18.5–24.9 (89.9%/94.3%) 25.0–29.9 (10.1%/5.7%)	N/A	Lin et al., 2017 [275]
miR-208a	Up	Plasma	RT-qPCR	CAD (290) HC (110)	Chinese	N/A	N/A	N/A	0.919 (0.893–0.945) 75.5; 93.6	Zhang et al., 2017 [278]
miR-133a	Up	Plasma	RT-qPCR	CAD (79) HC (63)	Chinese	58 ± 12 55 ± 11	57/22 39/24	24.8 ± 4.12 24.0 ± 3.7	0.597 (0.504–0.691) 29.1; 92.5	Zhu, 2017 [284]
miR-145-3p	Down	Serum	miSript miRNA PCR Array, RT-qPCR	CAD (40) HC (40)	Han Chinese	34.20 ± 5.93 36.58 ± 3.96	37/3 36/4	28.01 ± 4.90 27.33 ± 2.75	0.753 (0.643–0.863) 67.50; 82.10	Du et al., 2016 [254]
miR-190a-5p									0.782 (0.680–0.884) 70.00; 75.00	
miR-196b-5p									0.824 (0.731–0.917) 85.00; 72.50	
miR-3163-3p									0.758 (0.651–0.864) 57.50; 84.60	
miR-126-5p	Down	Plasma	qPCR	CAD (110) HC (40)	Chinese	66.5 ± 11.7 ^1a^ 67.4 ± 9.7 ^1b^ 68.9 ± 11.3 ^1c^ 64.0 ± 10.4	67/43 28/12	24.9 ± 2.7 ^1a^ 24.4 ± 3.0 ^1b^ 25.2 ± 3.2 ^1c^ 23.9 ± 3.5	N/A	Li et al., 2016 [285]
miR-208a, miR-370	Up	Plasma	RT-qPCR	CAD (95) HC (50)	Chinese	65 (44–78) 65 (46–75)	65/30 34/16	23 (20–26) 22 (20–24)	0.856 (0.796–0.917) ^2^ 73.7; 86.0	Liu et al., 2016 [270]
miR-15a-5p	Up	Plasma	RT-qPCR	CAD (50) HC (50)	Irish	65 ± 9 60 ± 13	43/7 42/8	27.69 ± 3.31 26.96 ± 2.96	0.67	O’Sullivan et al., 2016 [266]
miR-16-5p									0.68	
miR-93-5p									0.75	
miR-146a-5p	Down								0.65	
miR-206	Up	Plasma	Microarray, RT-qPCR	CAD (67) HC (67)	Chinese	64.70 ± 6.79 63.69 ± 5.96	43/24 32/35	N/A	0.607 (0.508–0.706)	Zhou et al., 2016 [255]
miR-574-5p									0.696 (0.609–0.787)	
miR-765	Up	Plasma	RT-qPCR	CAD (37) HC (20)	Chinese	72.97 ± 4.28 71.7 ± 5.2	25/12 10/10	23.08 ± 3.03 22.29 ± 1.49	0.959	Ali Sheikh et al., 2015 [272]
miR-149	Down								0.938	
miR-765	Up	Plasma	RT-qPCR	CAD (65) HC (32)	Chinese	53 (49–57) 53 (49–57)	38/27 16/16	22 (19–25) 22 (20–23)	0.968 (0.939–0.996) 81.5; 93.7	Ali Sheikh et al., 2015 [274]
miR-149	Down								0.938 (0.894–0.983) 71.8; 95.3	
miR-424									0.919 (0.863–0.975) 68.7; 92.3	
miR-17-5p	Up	Plasma	qPCR	CAD (59) NS-CAD (33) HC (20)	Chinese	65.07 ± 10.55 65.23 ± 7.46 55.90 ± 4.72	40/19 18/15 7/13	N/A	0.894 (0.780–0.968)	Chen et al., 2015 [280]
miR-145	Down	Plasma	RT-qPCR	CAD (26) HC (28)	Chinese	N/A	N/A	N/A	N/A	Gao et al., 2015 [286]
miR-21, miR-34a	Up	Plasma	Microarray (mice), RT-qPCR	CAD (32) HC (20)	Chinese	67 ± 11 62 ± 8	Only male	24.1 ± 3.7 23.6 ± 4.0	N/A	Han et al., 2015 [256]
miR-23a	Down									
miR-2909	Up	PBMC	RT-qPCR	CAD (80) HC (20)	Iranian	50 ± 4 49 ± 8	Only male	N/A	N/A	Arora et al., 2014 [277]
miR-208, miR-215, miR-487a, miR-502	Up	Serum	TLDA, RT-qPCR	CAD (92) HC (34)	Chinese	65.2 ± 10.5 59.4 ± 13.1	53/39 15/19	25.83 ± 1.48 24.82 ± 2.72	0.909 (0.858–0.960) ^2^ 83.7; 82.4	Wang et al., 2014 [257]
miR-29b	Down									
miR-1	Up	Plasma	Microarray, RT-qPCR	CAD (34) HC (20)	Italian	60.0 ± 10.6 62.5 ± 2.1	30/4 19/1	N/A	0.918	D’Alessandra et al., 2013 [258]
miR-126									0.929	
miR-485-3p									0.851	
miR-340, miR-624	Up	Platelet	Microarray, RT-qPCR	CAD (40) HC (40)	Dutch	51.4 ± 4.7 51.0 ± 4.6	Only male	N/A	0.71 (0.59–0.83) ^2^ (combined with miR-451, miR-454)	Sondermeijer et al., 2011 [259]
miR-133	Up	Plasma	RT-qPCR	CAD (36) HC (17)	German	67.69 ± 11.07 32.18 ± 8.78	25/11 6/11	> 25 (38.7%) > 25 (23.5%)	N/A	Fichtlscherer et al., 2010 [247]
miR-17, miR-92a, miR-126, miR-145, miR-155, miR-199a	Down									
miR-17, miR-92a, miR-126, miR-145, miR-155	Down	Serum		CAD (31) HC (14)		68.06 ± 9.66 39.28 ± 17.52	21/10 5/9	N/A		

^1a^ Syntax Score ≤ 22; ^1b^ Syntax Score > 22 and ≤ 32; ^1c^ Syntax Score > 32; ^2^ values for miRNA panel; ^3^ miRNA assessed for 70 patients from CAD and HC groups. miRNA—microRNA; CAD—coronary artery disease; HC—healthy controls; PBMC—peripheral blood mononuclear cell; qPCR—quantitative real-time polymerase chain reaction; RT-qPCR—reverse transcription quantitative real-time polymerase chain reaction; NGS—next-generation sequencing; sRNA-Seq—small RNA sequencing; TLDA—TaqMan Low Density Array; BMI—body mass index; AUC—area under the curve; 95% CI—95% confidence interval; SV—sensitivity; SP—specificity; NS—non-significant; N/A—not assessed.

## Data Availability

Not applicable.

## Abbreviations

ABCA1	ATP-binding cassette: sub-family A, member 1
ACS	acute coronary syndrome
ADAM10	a disintegrin and metalloproteinase domain-containing protein 10
AGEs	advanced-glycation end-products
AGO	Argonaute
Agr-1	argininase-1
AMPK	adenosine monophosphate-activated protein kinase
ApoA-1	apolipoprotein A-1
AUC	area under the curve
BMI	body mass index
CAC	coronary artery calcification
CAD	coronary artery disease
CAV-1	caveolin 1
CCL-2	chemokine CC-motif ligand 2
CCS	chronic coronary syndrome
CI	confidence interval
circRNA	circular RNA
COX	cyclooxygenase
DAXX	death-domain associated protein
DGCR8	DiGeorge syndrome Critical Region 8
EC	endothelial cell
ECM	extracellular matrix
EDCF	endothelium-derived cyclooxygenase-dependent contracting factor
EMP2	epithelial membrane protein 2
EndMT	endothelial-to-mesenchymal transition
eNOS	endothelial nitric oxide synthase
EPC	endothelial progenitor cell
ERK	extracellular signal-regulated kinase
ET-1	endothelin-1
EVs	extracellular vesicles
EXP5	Exportin-5
FSP-1	fibroblast specific protein-1
GLUT-4	glucose transporter-4
GS	Gensini score
HbA1c	glycated hemoglobin A1c
HDL-C	high-density lipoprotein cholesterol
HMGB1	high mobility group box-1
HSC70	heat shock cognate 70
HSP90	heat shock protein 90
ICAM-1	intercellular adhesion molecule-1
IGF-1R	insulin-like growth factor-1 receptor
IFN-γ	interferon-γ
IL	interleukin
IRAK1	interleukin 1 receptor associated kinase 1
IRS-1	insulin receptor substrate 1
JNK	c-Jun N-terminal kinase
KLF	Krüppel-like factor
KRAS	Kirsten Rat Sarcoma Viral Oncogene Homolog
LDL-C	low-density lipoprotein cholesterol
LNA	locked nucleic acid
lncRNA	long non-coding RNA
α-SMA	alpha smooth muscle actin
MAPK	mitogen-activated protein kinase
MCP-1	monocyte chemoattractant protein-1
MEK	mitogen-activated protein kinase
MGO	methylglyoxal
MPO	myeloperoxidase
mRNA	messenger RNA
miRNA, miR	microRNA
mTOR	mammalian target of rapamycin
NADPH	nicotinamide adenine dinucleotide phosphate
ncRNA	non-coding RNA
NF-κB	nuclear factor-kappa B
NGS	next-generation sequencing
NO	nitric oxide
NPM1	nucleophosmin 1
OR	odds ratio
ox-LDL	oxidized low-density lipoprotein
PAI-1	plasminogen activator inhibitor-1
PBMC	peripheral blood mononuclear cell
PDGF	platelet-derived growth factor
PDGFRβ	platelet-derived growth factor receptor beta
PECAM-1	platelet endothelial cell adhesion molecule-1
PHLPP2	PH domain leucine-rich repeat protein phosphatase 2
PI3K	phosphatidylinositol 3-kinase
piRNA	PIWI-interacting RNA
PKC	protein kinase C
pre-miRNA	precursor miRNA
pri-miRNA	primary miRNA
RAGE	receptor for advanced glycation end-products
RISC	RNA-induced silencing complex
RNA pol II	RNA polymerase II
ROC	receiver operating characteristic
ROCK1	Rho-associated coiled-coil forming protein kinase 1
ROS	reactive oxygen species
RT-qPCR	real-time polymerase chain reaction
siRNA	small interfering RNA
SM22α	smooth muscle protein 22 alpha
sncRNA	small non-coding RNA
Spred-1	sprouty-related, EVH1 domain-containing protein 1
sRAGE	soluble receptor for advanced glycation end-products
SREBP	sterol regulatory element-binding protein
SRF	serum-response factor
SYNTAX	Synergy between Percutaneous Coronary Intervention with Taxus and Cardiac Surgery
T2DM	type 2 diabetes mellitus
TC	total cholesterol
TG	triglycerides
TGF-β	transforming growth factor-β
TNF-α	tumor necrosis factor α
TPM1	tropomyosin 1
TRAF6	tumor necrosis factor receptor associated factor 6
TRBP	trans-activation response RNA binding protein
3′ UTR	3′ untranslated region
WHR	waist-to-hip ratio
VCAM-1	vascular cell adhesion molecule-1
VE-cadherin	vascular endothelial cadherin
VEGF	vascular endothelial growth factor
VSMC	vascular smooth muscle cell
vWF	von Willebrand Factor
